# Endocytic protein intersectin1-S shuttles into nucleus to suppress the DNA replication in breast cancer

**DOI:** 10.1038/s41419-021-04218-1

**Published:** 2021-10-08

**Authors:** Huikun Zhang, Zhifang Guo, Xiaoli Liu, Yawen Zhao, Yongzi Chen, Ming Zhang, Li Fu, Feng Gu, Yongjie Ma

**Affiliations:** 1 Department of Tumor Cell Biology, Tianjin Medical University Cancer Institute and Hospital, National Clinical Research Center for Cancer, Tianjin, China; 2 Tianjin’s Clinical Research Center for Cancer, Tianjin Medical University Cancer Institute and Hospital, Tianjin, China; 3 Key Laboratory of Cancer Prevention and Therapy, Tianjin, China; 4 Key Laboratory of Breast Cancer Prevention and Therapy, Tianjin Medical University, Ministry of Education, Tianjin, China; 5 Department of Epidemiology and Biostatistics, Institute of Bioinformatics, University of Georgia, Athens, GA USA; 6 Department of Breast Cancer Pathology and Research Laboratory, Tianjin Medical University Cancer Institute and Hospital, Tianjin, China

**Keywords:** Cancer, Cell biology

## Abstract

Breast cancer is the most common type of cancer worldwide. However, the well-known molecular biomarkers are not enough to meet the needs of precision medicine. In search for novel targets in this regard, we reported ITSN1 (intersectin1) as one of the candidates through mRNA microarray analysis. In the present study, we reported that endocytic protein ITSN1-S exists not only in the cytoplasm but also in nuclei of breast cancer cells. ITSN1-S′ functional nuclear localization signal is within its residues 306–312. Its nuclear export signal (NES) resides within its SH3 domains. We also found, the interaction between the CC domain of nuclear ITSN1-S and the NT domain of nuclear DNA helicase II (NDH II) directly suppressed the DNA replication and nascent DNA synthesis by inhibiting the R-loops resolution in breast cancer cells. Furthermore, the interaction between the EH domains of cytoplasmic ITSN1-S and PI3KC2α inhibit cell migration and invasion by inactivating the PI3KC2α-AKT pathway. Our results were confirmed in both *ITSN1* gene knockout cells and in vivo assays. Finally, our clinical data showed a potential application of the combined consideration of the cytoplasmic and nuclear ITSN1-S as an independent prognosis factor. In conclusion, our study revealed ITSN1-S′ novel positioning in the nuclei of breast cancer cells, its function in suppressing DNA replication, and its potential application in improved breast cancer prognosis.

## Introduction

The incidence and mortality of breast cancer continue to increase and has become the most commonly diagnosed cancer worldwide in 2020 [1, 2]. However, the well-known molecular biomarkers are not enough to meet the needs of precision medicine [3]. Novel targets for improved prognosis are urgently needed. In this regard, we identified ITSN1 (intersectin1) as one of the candidates through the mRNA microarray analyses on the invasive ductal carcinoma (IDC) tissues with the paired adjacent tissues from 22 patients and 34 patients, respectively.

ITSN1 is a multi-domain adapter protein that participates in endocytosis, exocytosis, and cell signaling in the cytoplasm [4–6]. ITSN1 consists of two isoforms, a long isoform (ITSN1-L) and a short isoform (ITSN1-S) [7, 8]. ITSN1-S is expressed ubiquitously, while ITSN1-L is specifically expressed in neurons. Our previous studies showed that ITSN1-S promoted tumor development in malignant glioma [5, 9]. Russo A et al. reported a similar conclusion in neuroblastoma [10]. On the contrary, ITSN1-S was reported to exert inhibitory function on proliferation and metastatic abilities of human lung cancer [11]. Our present study focused on the role of ITSN1-S in breast cancer and our bioinformatics analysis from an online database showed that ITSN1 expression was downregulated in breast cancer tissues and its expression was positively associated with patient’s survival, suggesting ITSN1 may play a suppressive role in tumorigenesis of breast cancer.

Our present study reported that ITSN1-S was expressed in IDC tissues. Furthermore, both in vitro and in vivo experiments confirmed that ITSN1-S was located in the cytoplasm and nuclei. We also identified the nuclear localization signal and NES of ITSN1-S. ITSN1-S shuttled into the nucleus to suppress the DNA replication. Finally, our clinical data showed a potential application of the combined consideration of the cytoplasmic and nuclear ITSN1-S as an independent prognosis factor. Taken together, our study revealed ITSN1-S′ novel positioning in the nuclei of breast cancer cells, its function in suppressing DNA replication, and its potential application in improved breast cancer prognosis.

## Results

### Endocytic protein ITSN1-S localized not only in the cytoplasm but also in nuclei of breast cancer cells

**Fig. 1:**
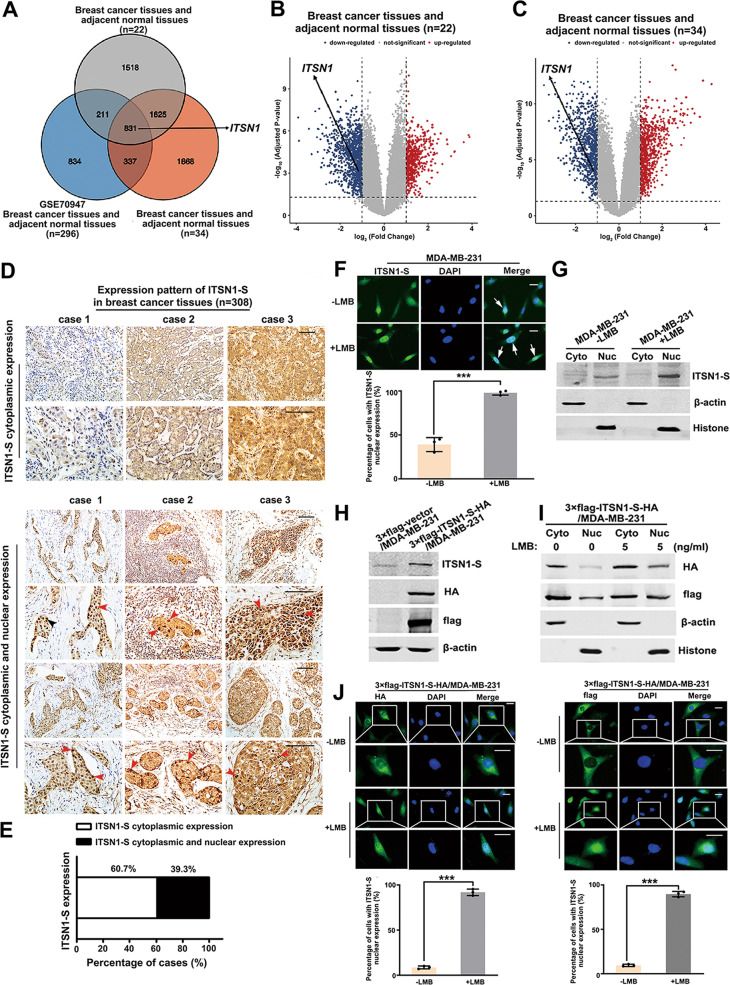
ITSN1-S localized not only in the cytoplasm but also in nuclei of breast cancer tissues and cells. **A** Venn diagram showed the DEGs in breast cancer tissues and their paired adjacent tissues by combination analysis of our microarray data from 22 patients’ samples and 34 patients’ samples with the mRNA expression profile of breast cancer patients’ samples (*n* = 296) downloaded from GEO (ID: GSE70947). Genes with *P* < 0.05 and |Fold Change | >1.5 were considered as DEGs. **B**, **C** Volcano plots showed the DEGs from our microarray analyses of 22 paired breast adjacent normal/tumor tissues (**B**) and 34 paired breast adjacent normal/tumor tissues (**C**). The red dots and blue dots represented the upregulated and downregulated genes. X-axes showed log_2_ | Fold change| and y-axes showed -log_10_(*P* value). **D** Expression pattern of ITSN1-S in breast cancer tissues (*n* = 308). Top: representative images of ITSN1-S cytoplasmic expression in IDC specimens. Bottom: representative images of ITSN1-S cytoplasmic and nuclear expression in IDC specimens. Scale bars, 100 μm. **E** Among 308 IDC cases, 60.7% (187/308) cases showed ITSN1-S cytoplasmic positive expression, 39.3% (121/308) cases showed ITSN1-S cytoplasmic and nuclear positive expression. **F** Representative immunofluorescence images of endogenous ITSN1-S localization in MDA-MB-231 cells treated with 1 ng/ml LMB for 12 h (+LMB) or without LMB as a control (−LMB). Arrowheads showed the cells with nuclear colocalization. Quantitative results were analyzed in the lower panel. Values were expressed as mean ± SD from three independent experiments (two-tailed Student’s *t*-test, ^***^P < 0.001). **G** Western blots analysis of endogenous ITSN1-S expression in the cytoplasm (Cyto) and nuclei (Nuc) of MDA-MB-231 cells. β-actin and histone were used as specific markers for cytoplasm and nuclei, respectively. **H** Western blot of ITSN1-S expression in 3×flag-ITSN1-S-HA/MDA-MB-231 cells. ITSN1-S expression was detected by anti-ITSN1-S, anti-flag, anti-HA antibodies. **I** Western blot analysis of exogenous ITSN1-S in the cytosol (Cyto) and nuclear (Nuc) of 3×flag-ITSN1-S-HA/MDA-MB-231 cells. β-actin and histone were used as specific markers for cytoplasm and nuclei, respectively. **J** Exogenous HA and 3×flag-labeled ITSN1-S protein was detected by immunofluorescence with anti-HA (left panel) and anti-flag (right panel) antibodies in 3×flag-ITSN1-S-HA/MDA-MB-231 cells treated with 5 ng/ml LMB for 5 h (+LMB) or without LMB as a control (−LMB). Scale bars, 25 μm. Quantitative results were analyzed in the lower panel. Values were expressed as mean ± SD from three independent experiments (two-tailed Student’s *t*-test, ^***^P < 0.001).

In order to identify differentially expressed genes (DEGs) involved in breast cancer progression, we applied mRNA microarray to generate specific sets of genes at genome-wide in IDC tissues with their paired adjacent tissues from 22 patients’ samples and 34 patients’ samples, respectively. Fig 1A showed 831 overlapped DEGs, including ITSN1, by combination analysis of our microarray data with the mRNA expression profile of breast cancer tissues downloaded from GEO (GSE70947). Volcano plots showed that the mRNA level of ITSN1 was lower in breast cancer tissues compared with adjacent normal tissues (Fig. 1B, C). It suggested an important role of ITSN1 in the suppression of breast cancer progression. Then we performed immunohistochemistry (IHC) to examine the localization of ITSN1-S, the major isoform of ITSN1 in breast cancer in breast cancer tissues [12]. IHC analysis showed that ITSN1-S localized not only in the cytoplasm but also in cells nuclei (Fig. 1D). Cytoplasmic ITSN1-S expression was observed in 60.7% of IDC cases (187/308). About 39.3% of cases (121/308) showed both cytoplasmic and nuclear expression (Fig. 1E). Then the subcellular localization of ITSN1-S was also confirmed in cells. About 32.3% of cells with nuclear ITSN1-S expression were observed in MDA-MB-231 cells. The colocalization of nuclei and ITSN1-S was further validated by treatment with LMB (a specific inhibitor of the nuclear export receptor CRM1) [13] (Fig. 1F). Western blot analysis showed similar results (Fig. 1G). Then, cell clones overexpressing 3×flag and HA-labeled ITSN1-S were applied to indicate the nuclear localization (Fig. 1H). Nuclear/cytosol fractionation assay and immunofluorescence analysis showed that exogenous ITSN1-S mainly localized in the cytoplasm of MDA-MB-231 cells, while exogenous ITSN1-S accumulated in the nucleus upon LMB treatment (Fig. 1I, J). It suggested that ITSN1-S as a nucleocytoplasmic trafficking protein in a CRM1-dependent pathway.

### ITSN1-S contained a nuclear localization signal in residues 306–312

**Fig. 2:**
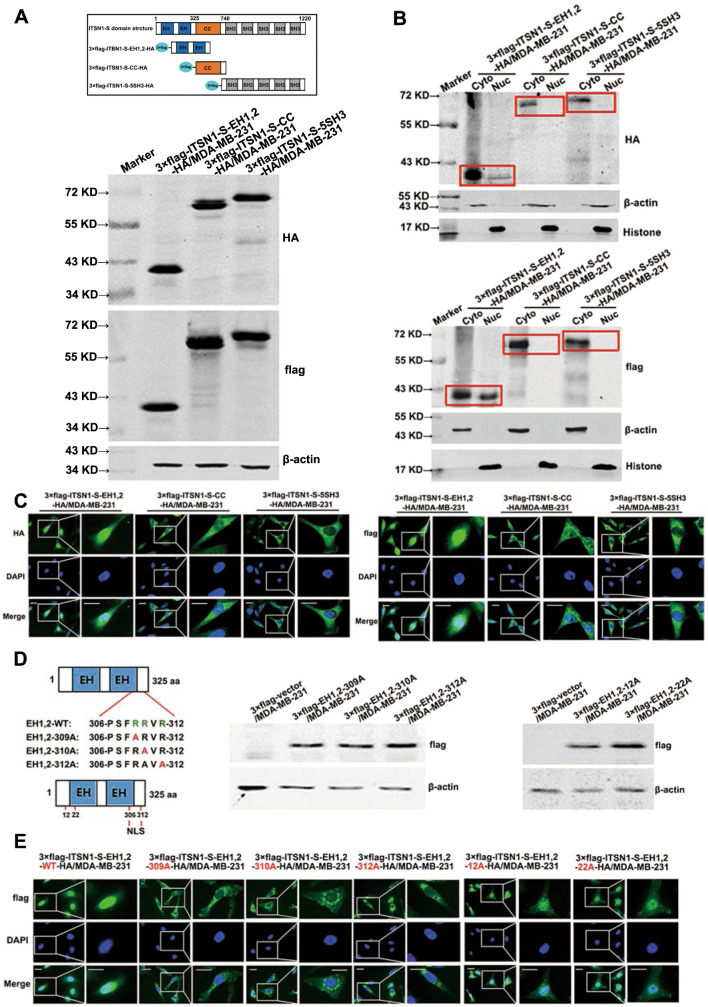
ITSN1-S contained a nuclear localization signal (NLS) in resides 306–312. **A** Various fragments of ITSN1-S labeled with flag and HA tags were transfected into MDA-MB-231 cells. Top: domain structure of human ITSN1-S. Bottom: expression of exogenous fragments (EH1,2, CC, and 5SH3) were monitored by anti-HA and anti-flag antibodies in Western blot analysis, respectively. β-actin was used as a loading control. **B** Expression of exogenous fragments of ITSN1-S in the cytoplasm (Cyto) and nuclei (Nuc) of MDA-MB-231 cells were detected by anti-HA and anti-flag antibodies in Western blot analysis, respectively. **C** Localization of exogenous ITSN1-S fragments were detected by immunofluorescence with anti-HA (left panel) and anti-flag (right panel) antibodies in MDA-MB-231 cells, respectively. Scale bars, 25 μm. **D** Left: schematic presentation of the different NLS mutants of ITSN1-S. The mutated residues were shown in red. Right: expression of 3×flag-labeled mutated NLSs were detected by anti-flag antibody in Western blot analysis. β-actin was used as a loading control. **E** Localization of 3×flag-labeled mutated NLSs were detected by immunofluorescence with anti-flag antibody in MDA-MB-231 cells. Scale bars, 25 μm.

Most macromolecules contain nuclear localization signals (NLSs) and NESs for nuclear import and export [14, 15]. Considering the molecular mass of ITSN1-S protein, ITSN1-S may contain a functional NLS. To identify the NLS of ITSN1-S, lentivirus-expressing 3×flag and HA-labeled ITSN1-S fragments (EH1,2, CC, and 5SH3) were transfected into MDA-MB-231 cells, respectively (Fig. 2A). Nuclear/cytosol fractionation assay showed that EH domains (EH1, 2) were detected in both nucleus and cytoplasm, whereas coiled-coil (CC) domain and SH3 domains (5SH3) were mainly localized in the cytoplasm (Fig. 2B). Immunofluorescence analysis showed similar results (Fig. 2C). It suggested that EH domains of ITSN1-S may contain an NLS. Then we searched the potential NLS sequence of ITSN1-S protein which is specifically localized in the EH domains by using the online tool (https://psort.hgc.jp/form2.html). According to the results, ITSN1-S contained a conserved bipartite NLS in residues 306–312 in EH domains. To reveal whether the conserved bipartite NLS in ITSN1-S is functional and is responsible for the entry of ITSN1-S into the nucleus, mutation analysis was performed. Several NLS mutants were constructed with different arginine to alanine residue substitutions (Fig. 2D). The cellular localization of the above NLS mutants were detected by immunofluorescence analysis. Our results showed that substitutions of arginine to alanine residues at positions 309, 310, or 312, but not positions 12 or 22 (which were not in the potential NLS region and regarded as negative controls), failed to enter the nucleus (Fig. 2E). These results suggested that residues 306–312 (PSFRRVR) constituted a functional NLS in ITSN1-S protein.

### NES of ITSN1-S located within its SH3 domains

**Fig. 3:**
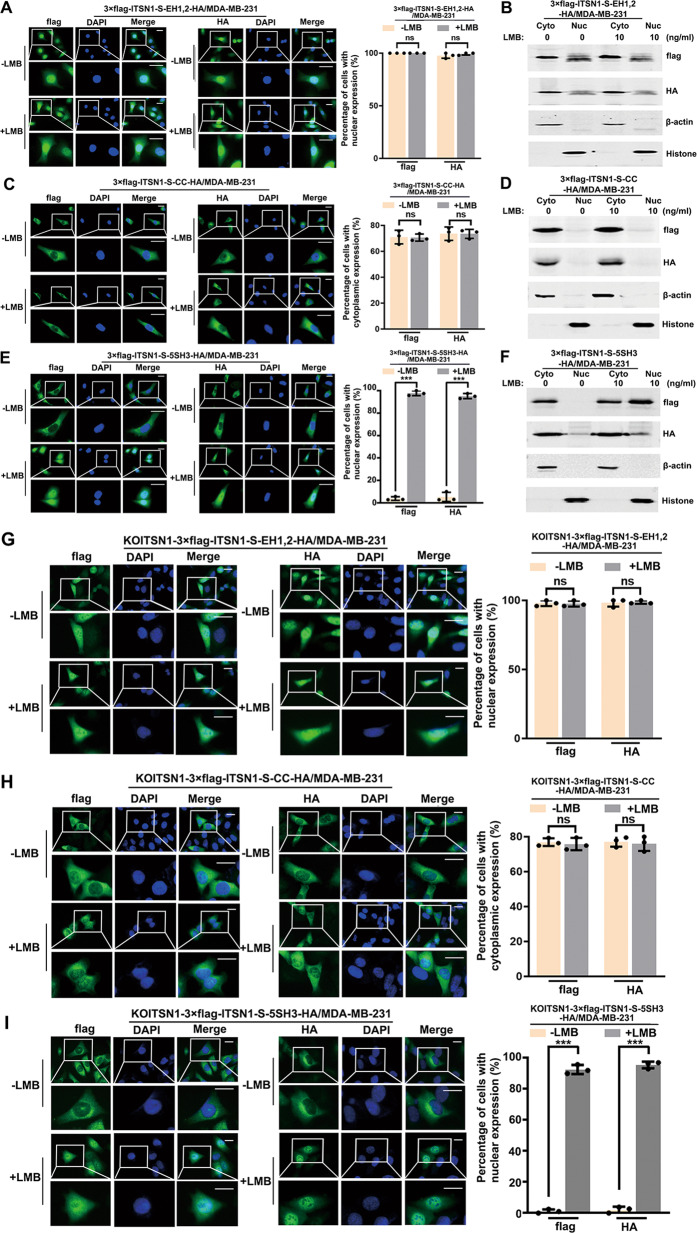
Nuclear export signal (NES) of ITSN1-S located within its SH3 domains. **A** Representative immunofluorescence images of MDA-MB-231 cells treated with 10 ng/ml LMB for 5 h (+LMB) or without LMB as a control (−LMB). Localization of EH domains (EH1,2) of ITSN1-S was detected using anti-flag and anti-HA antibodies, respectively. Scale bars, 25 μm. Quantitative results were analyzed in the right panel. Values were expressed as mean ± SD from three independent experiments (two-tailed Student’s *t*-test). ns, no significance. **B** Expression of EH domains of ITSN1-S in the cytoplasm (Cyto) and nuclei (Nuc) was detected by Western blot analysis in MDA-MB-231 cells treated with 10 ng/ml LMB for 5 h or without LMB as a control. β-actin and histone were used as specific markers for cytoplasm and nuclei, respectively. **C** Representative immunofluorescence images of MDA-MB-231 cells treated with 10 ng/ml LMB for 5 h (+LMB) or without LMB as a control (−LMB). Localization of the CC domain of ITSN1-S was detected using anti-flag and anti-HA antibodies, respectively. Scale bars, 25 μm. Quantitative results were analyzed in the right panel. Values were expressed as mean ± SD from three independent experiments (two-tailed Student’s *t*-test). ns, no significance. **D** Expression of CC domain of ITSN1-S in the cytoplasm (Cyto) and nuclei (Nuc) was detected by Western blot analysis in MDA-MB-231 cells treated with 10 ng/ml LMB for 5 h or without LMB as a control. β-actin and histone were used as specific markers for cytoplasm and nuclei, respectively. **E** Representative immunofluorescence images of MDA-MB-231 cells treated with 10 ng/ml LMB for 5 h (+LMB) or without LMB as a control (−LMB). Localization of SH3 domains (5SH3) of ITSN1-S was detected using anti-flag and anti-HA antibodies, respectively. Scale bars, 25 μm. Quantitative results were analyzed in the right panel. Values were expressed as mean ± SD from three independent experiments (two-tailed Student’s *t*-test, ^***^P < 0.001). **F** Expression of SH3 domains of ITSN1-S in the cytoplasm (Cyto) and nuclei (Nuc) was detected by Western blot analysis in MDA-MB-231 cells treated with 10 ng/ml LMB for 5 h or without LMB as a control. β-actin and histone were used as specific markers for cytoplasm and nuclei, respectively. **G** Representative immunofluorescence images of KOITSN1/MDA-MB-231 cells treated with 10 ng/ml LMB for 5 h (+LMB) or without LMB as a control (−LMB). Localization of EH domains (EH1,2) of ITSN1-S was detected using anti-flag and anti-HA antibodies, respectively. Scale bars, 25 μm. Quantitative results were analyzed in the right panel. Values were expressed as mean ± SD from three independent experiments (two-tailed Student’s *t*-test). ns, no significance. **H** Representative immunofluorescence images of KOITSN1/MDA-MB-231 cells treated with 10 ng/ml LMB for 5 h (+LMB) or without LMB as a control (−LMB). Localization of CC domains (CC) of ITSN1-S was detected using anti-flag and anti-HA antibodies, respectively. Scale bars, 25 μm. Quantitative results were analyzed in the right panel. Values were expressed as mean ± SD from three independent experiments (two-tailed Student’s *t*-test). ns, no significance. **I** Representative immunofluorescence images of KOITSN1/MDA-MB-231 cells treated with 10 ng/ml LMB for 5 h (+LMB) or without LMB as a control (−LMB). Localization of SH3 domains (5SH3) of ITSN1-S was detected using anti-flag and anti-HA antibodies, respectively. Scale bars, 25 μm. Quantitative results were analyzed in the right panel. Values were expressed as mean ± SD from three independent experiments (two-tailed Student’s *t*-test, ^***^P < 0.001).

In order to find the mechanism responsible for the exit of ITSN1-S from the nucleus, cell clones overexpressing 3×flag and HA-labeled ITSN1-S fragments were treated with or without LMB, respectively. Immunofluorescence analysis showed the addition of LMB did not affect the subcellular localization of EH domains in MDA-MB-231 cells (Fig. 3A). The Nuclear/cytosol fractionation assay confirmed the results (Fig. 3B). Similarly, the CC domain is always mainly retained in the cytoplasm of MDA-MB-231 cells with or without LMB treatment (Fig. 3C, D). Meanwhile, SH3 domains presented in the cytoplasm without LMB treatment while it mainly accumulated in the nucleus of LMB-treated MDA-MB-231 cells (Fig. 3E, F). Then CRISPR/Cas9 system was used to generate *ITSN1* gene knockout cells (KOITSN1/MDA-MB-231) (Supplementary Fig. S1A). KOITSN1/MDA-MB-231 cell clones overexpressing 3×flag and HA-labeled ITSN1-S fragments were constructed (Supplementary Fig. S1B). The results in KOITSN1/MDA-MB-231 cells were similar to MDA-MB-231 cells (Fig. 3G–I). It suggested that SH3 domains seemed to be necessary for ITSN1-S cytoplasmic localization. However, this region did not have a canonical NES. Taken together, ITSN1-S shuttled between the nucleus and cytoplasm, its import into the nucleus depended on an NLS and the NES may locate within its SH3 domains.

### ITSN1-S in the nucleus inhibited breast cancer cells proliferation in vitro and in vivo

**Fig. 4:**
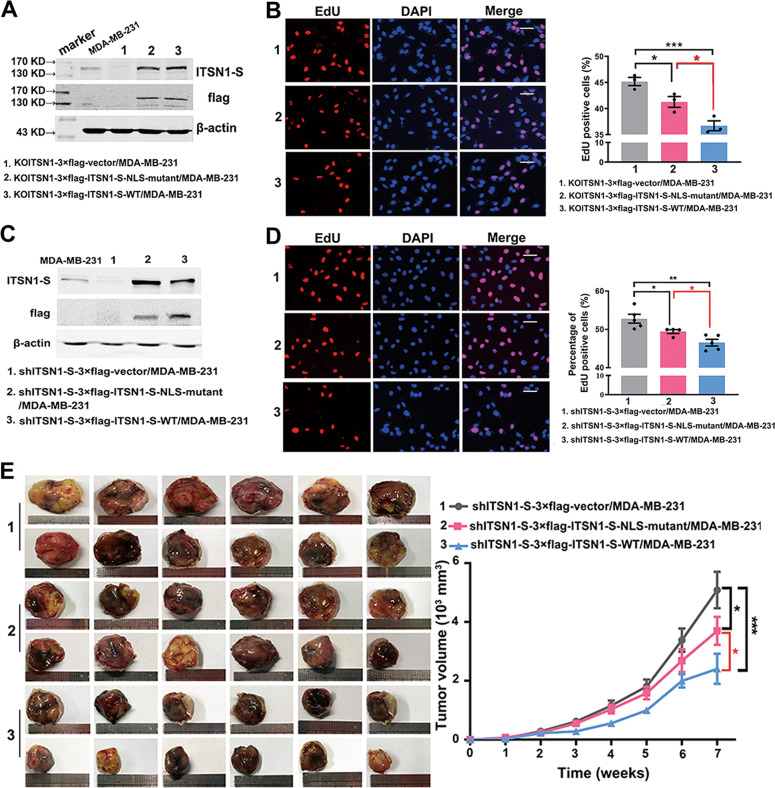
ITSN1-S in nucleus inhibited breast cancer cells proliferation in vitro and in vivo. **A** 3×flag-labeled vector, 3×flag-labeled NLS-mutant ITSN1-S, and 3×flag-labeled wild-type ITSN1-S were transfected into KOITSN1/MDA-MB-231 cell clones and tested with anti-flag and anti-ITSN1-S antibodies in Western blot, respectively. β-actin was used as a loading control. **B** Proliferation ability was examined by EdU incorporation assay in KOITSN1–3×flag-vector/MDA-MB-231, KOITSN1–3×flag-ITSN1-S-NLS-mutant/MDA-MB-231, and KOITSN1–3×flag-ITSN1-S-WT/MDA-MB-231 cells. Scale bars, 25 μm. Quantitative results were analyzed in the lower panel. Values were expressed as mean ± SD from three independent experiments (two-tailed Student’s *t*-test, ^*^P < 0.05, ^***^P < 0.001). **C** 3×flag-labeled vector, 3×flag-labeled NLS-mutant ITSN1-S (which accumulated in the cytoplasm), and 3×flag-labeled wild-type ITSN1-S (which accumulated in both cytoplasm and nucleus) were transfected into shITSN1-S #2/MDA-MB-231 cell clones and tested with anti-flag and anti-ITSN1-S antibodies in Western blot, respectively. β-actin was used as a loading control. **D** Proliferation ability was examined by EdU incorporation assay in shITSN1-S-3×flag-vector/MDA-MB-231, shITSN1-S-3×flag-ITSN1-S-NLS-mutant/MDA-MB-231, and shITSN1-S-3×flag-ITSN1-S-WT/MDA-MB-231 cells. Scale bars, 25 μm. Quantitative results were analyzed in the right panel. Values were expressed as mean ± SD from three independent experiments (two-tailed Student’s *t*-test, ^*^P < 0.05, ^**^P < 0.01). **E** Orthotopic xenograft models were performed in vivo. The representative images of tumor size of shITSN1-S-3×flag-vector/MDA-MB-231 (*n* = 18), shITSN1-S-3×flag-ITSN1-S-NLS-mutant/MDA-MB-231 (*n* = 16), and shITSN1-S-3×flag-ITSN1-S-WT/MDA-MB-231 (*n* = 18) mice groups were presented in the left panel. Quantitative results were analyzed in the right panel. Values were expressed as mean ± SEM (two-way ANOVA, ^*^P < 0.05, ^***^P < 0.001).

We next focused on how nuclear ITSN1-S contributed to breast cancer progression. Plasmids overexpressing 3×flag-vector, 3×flag-labeled NLS-mutant ITSN1-S (which accumulated in the cytoplasm), and 3×flag-labeled wild-type ITSN1-S (which accumulated in both cytoplasm and nucleus) were transfected into KOITSN1/MDA-MB-231 cell clones, respectively (Fig. 4A). EdU incorporation assay showed that both NLS-mutant ITSN1-S (cytoplasmic ITSN1-S) and wild-type ITSN1-S (cytoplasmic and nuclear ITSN1-S) led to lower proliferation capacity than control, and wild-type ITSN1-S exhibited lower proliferation capacity than NLS-mutant ITSN1-S, suggesting that ITSN1-S in the nucleus could inhibit cell proliferation (Fig. 4B). Next, ITSN1-S expression was knocked down by five different shRNAs in MDA-MB-231 cells. ITSN1-S protein and mRNA expression levels were most significantly reduced in the #2 cell clone (Supplementary Fig. S1C). Plasmids overexpressing 3×flag-labeled NLS-mutant ITSN1-S (which cannot be shuttled into the nucleus) or 3×flag-labeled wild-type ITSN1-S (which accumulated in both cytoplasm and nucleus) were then transfected into shITSN1-S #2/MDA-MB-231 cell clones, named as shITSN1-S-3×flag-ITSN1-S-NLS-mutant/MDA-MB-231 and shITSN1-S-3×flag-ITSN1-S-WT/MDA-MB-231, respectively. shITSN1-S-3×flag-vector/MDA-MB-231 cells (which exhibited very few nuclear and cytosolic ITSN1-S distribution) were designed as control (Fig. 4C). EdU incorporation assay in shITSN1-S #2/MDA-MB-231 cells showed similar results with KOITSN1/MDA-MB-231 cells (Fig. 4D). Then the roles of nuclear ITSN1-S in breast cancer cells were confirmed in vivo. The tumor volume was decreased in NLS-mutant ITSN1-S (shITSN1-S-3×flag-ITSN1-S-NLS-mutant/MDA-MB-231) and wild-type ITSN1-S (shITSN1-S-3×flag-ITSN1-S-WT/MDA-MB-231) mice groups compared with the control (shITSN1-S-3×flag-vector/MDA-MB-231 mice group), and wild-type ITSN1-S mice group exhibited the minimum xenografts (Fig. 4E). This further demonstrated that ITSN1-S in the nucleus inhibited breast cancer cells proliferation.

### ITSN1-S suppressed nascent DNA synthesis of breast cancer cells. ITSN1-S could interact with NDH II and co-localize in the nucleus

**Fig. 5:**
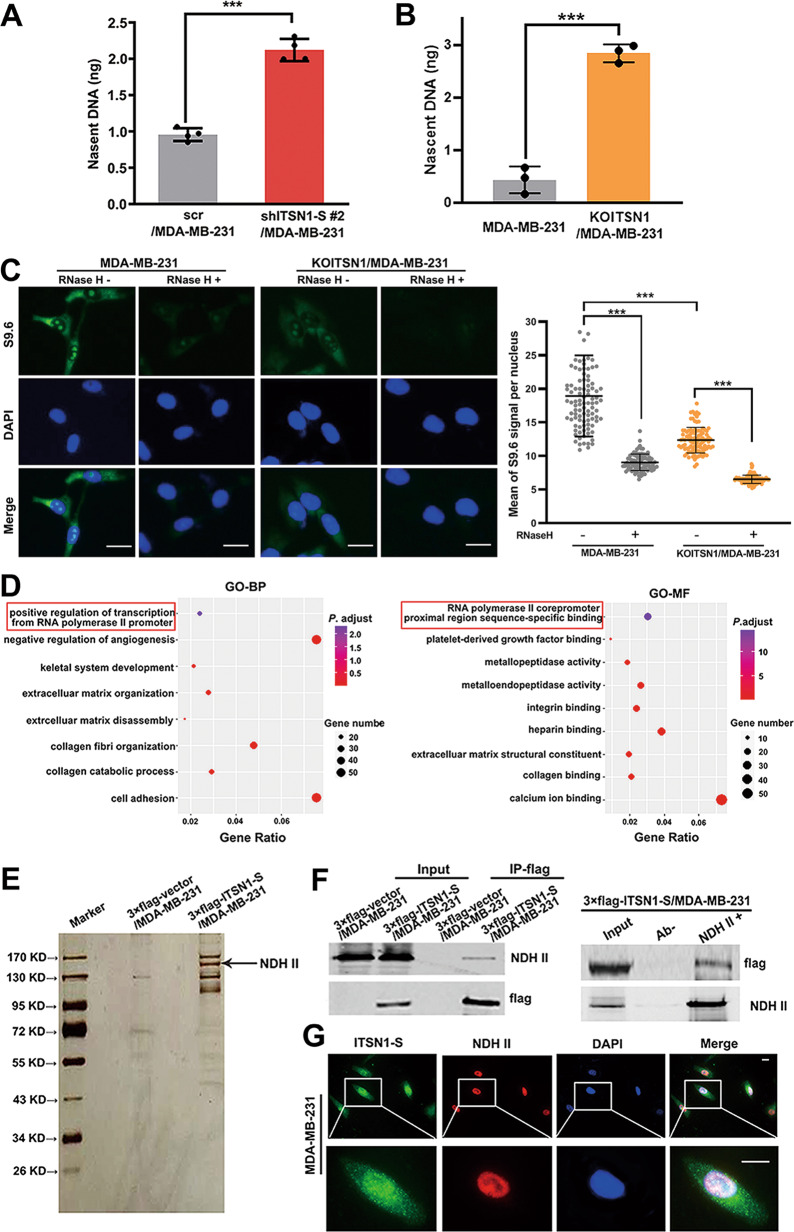
ITSN1-S suppressed nascent DNA synthesis of breast cancer cells. ITSN1-S could interact with NDH II and co-lcalize in the nucleus. **A** Quantification by qPCR of nascent DNA abundance in shITSN1-S #2/MDA-MB-231 and control cells. Values were expressed as mean ± SD from four independent experiments (two-tailed Student’s *t*-test, ^***^P < 0.001). **B** Quantification by qPCR of nascent DNA abundance in KOITSN1/MDA-MB-231 and control cells. Values were expressed as mean ± SD from three independent experiments (two-tailed Student’s *t*-test, ^***^P < 0.001)^.^ **C** R-loops were detected by immunofluorescence with the anti-RNA/DNA hybrid antibody S9.6 in KOITSN1/MDA-MB-231 and control cells. Pretreatment with RNase H for 12 h was used as a negative control. Scale bars, 25 μm. Quantitative results were analyzed in the right panel (two-tailed Student’s *t*-test, ^***^P < 0.001). **D** GO pathway analysis of the 759 DEGs by DAVID database. BP biological progress, MF molecular function. Y-axes showed the GO terms and x-axes showed the gene ratio of each term. **E** Mass spectrometric analysis of 3×flag-ITSN1-S-associated proteins. **F** IP experiments were performed by using an anti-flag M2 affinity gel (left panel), antibodies against NDH II or control IgG (right panel), respectively. Expression of flag-tagged ITSN1-S and NDH II was determined by Western blot analysis. **G** Representative immunofluorescent images of the colocalization of ITSN1-S and NDH II in MDA-MB-231 cells. Scale bars, 25 μm.

In order to further investigate the role of nuclear ITSN1-S in proliferation and DNA replication, we examined whether ITSN1-S suppression affected the synthesis of new (“nascent”) DNA [16]. Nascent DNA abundance was quantitated at the *c-MYC* gene origin in MDA-MB-231 cells. It showed that loss of ITSN1-S resulted in a twofold increase in the abundance of nascent DNA (Fig. 5A). Deletion of ITSN1 increased nascent DNA abundance in MDA-MB-231 cells (Fig. 5B). We next investigated how ITSN1-S promoted nascent DNA synthesis. RNA displacement loops (R-loops) consist of an RNA/DNA hybrid and a displaced non-template DNA strand [17–19]. Emerging evidence has shown that persistent R-loops make the genome vulnerable to DNA damage due to exposure of ssDNA regions and blockage of replication fork progression, leading to replication stress [5, 20]. Then immunostaining assays with S9.6 antibody were performed to detect R-loops in cells. The S9.6 antibody is broadly used to detect RNA:DNA hybrids, while using the S9.6 antibody for imaging can be problematic because it readily binds to double-strand RNA, giving rise to nonspecific signal [21]. We applied RNase H pretreatment as a negative control in the experiments [22]. Figure 5C showed that depletion of ITSN1 decreased the intensity of the S9.6 signal without RNase H pretreatment, which was abolished by pretreatment of RNase H. It suggested that ITSN1 in the nucleus could inhibit R-loops resolution or increase R-loops formation, leading to suppressed DNA replication and decreased nascent DNA synthesis. To further gain insights into the mechanism of the suppression role of nuclear ITSN1-S in DNA replication, the analysis of 817 breast cancer patient’s RNA-seq data from The Cancer Genome Atlas (TCGA) was performed to explore the functions of ITSN1 in breast carcinoma. About 759 DEGs which were detected between high and low ITSN1 expression patients were enriched by using the DAVID database for Gene Ontology (GO) functional enrichment analysis. Go analysis suggested that DEGs were enriched in several biological process (BP) and molecular function (MF) terms, such as positive regulation of transcription from RNA polymerase II promoter and RNA polymerase II promoter-proximal region sequence-specific binding (Fig. 5D). We found that NDH II, also named as ATP-dependent RNA helicase A and participated in the above functions, was one of the interaction proteins of nuclear ITSN1-S (Fig. 5E). Our results revealed that ITSN1-S and NDH II could co-immunoprecipitate and co-localize in the nucleus (Fig. 5F, G).

### The interaction between the CC domain of ITSN1-S and NT domain of NDH II directly suppressed the DNA replication and nascent DNA synthesis by inhibiting R-loops resolution in breast cancer cells

**Fig. 6:**
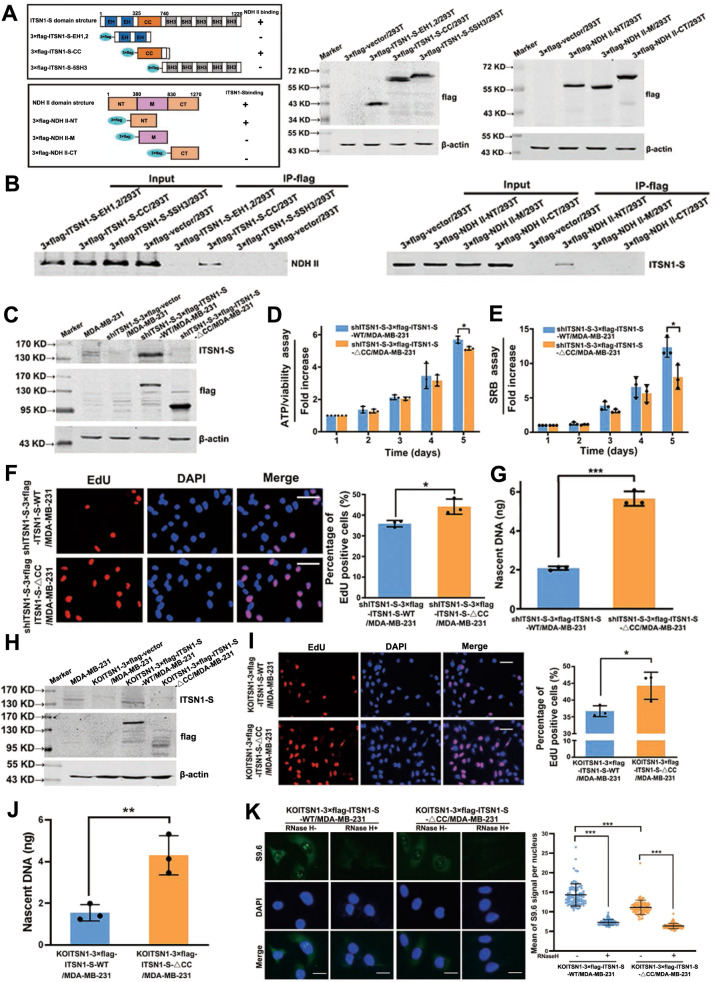
The interaction between the CC domain of ITSN1-S and the NT domain of NDH II directly suppressed DNA replication and nascent DNA synthesis by inhibiting R-loops resolution in breast cancer cells. **A** Several exogenous different domain structure fragments of NDH II or ITSN1-S were transfected into HEK-293T cells, respectively. Expression of exogenous fragments was monitored by anti-flag antibody in Western blot analysis. **B** Cellular extracts of the above cells were applied, and IP was performed by an anti-flag M2 affinity gel. Expression of ITSN1-S or NDH II was determined by Western blot analysis. **C** 3×flag-vector, 3×flag-ITSN1-S, and 3×flag-ITSN1-S-△CC were transfected into shITSN1-S#2/MDA-MB-231 cells and tested with anti-flag and anti-ITSN1-S antibodies by Western blot. β-actin was used as a loading control. **D**–**F** Proliferation ability was examined by ATP/viability assay (**D**), SRB assay (**E**), and EdU incorporation assays (**F**) in shITSN1-S-3×flag-ITSN1-S-WT/MDA-MB-231 and shITSN1-S-3×flag-ITSN1-S-△CC/MDA-MB-231 cells. Values were expressed as mean ± SD from three independent experiments (two-tailed Student’s *t*-test, ^*^P < 0.05). Scale bars, 25 μm. **G** Quantification by qPCR of nascent DNA abundance of indicated cells. Values were expressed as mean ± SD from three independent experiments (two-tailed Student’s *t*-test, ^***^P < 0.001). **H** 3×flag-vector, 3×flag-ITSN1-S, and 3×flag-ITSN1-S-△CC were transfected into KOITSN1/MDA-MB-231 cells and tested with anti-flag and anti-ITSN1-S antibodies by Western blot. β-actin was used as a loading control. **I** Proliferation ability was examined by EdU incorporation assay in KOITSN1–3×flag-ITSN1-S-WT/MDA-MB-231 and KOITSN1–3×flag-ITSN1-S-△CC/MDA-MB-231 cells. Scale bars, 25 μm. Quantitative results were analyzed in the right panel. Values were expressed as mean ± SD from three independent experiments (two-tailed Student’s *t*-test, ^*^P < 0.05). **J** Quantification by qPCR of nascent DNA abundance in KOITSN1–3×flag-ITSN1-S-WT/MDA-MB-231 and KOITSN1–3×flag-ITSN1-S-△CC/MDA-MB-231 cells. Values were expressed as mean ± SD from three independent experiments (two-tailed Student’s *t*-test, ^**^P < 0.01). **K** R-loops were detected by immunofluorescence with the anti-RNA/DNA hybrid antibody S9.6 in KOITSN1–3×flag-ITSN1-S-WT/MDA-MB-231 and KOITSN1–3×flag-ITSN1-S-△CC/MDA-MB-231 cells. Pretreatment with RNase H for 12 h was used as a negative control. Scale bars, 25 μm. Quantitative results were analyzed in the right panel (two-tailed Student’s *t*-test, ^*^P < 0.05).

NDH II unwinds DNA and RNA in a 3′ to 5′ direction and plays important roles in many processes, such as DNA replication, transcriptional activation, posttranscriptional RNA regulation, mRNA translation, and RNA-mediated gene silencing [16, 23, 24]. Therefore, we hypothesized whether the ITSN1-S/NDH II interaction in the nucleus affected DNA replication. Next, lentivirus-expressing 3×flag and HA-labeled NDH II fragments (NT, M, and CT) and ITSN1-S fragments (EH1,2, CC, and SH3) were transfected into HEK-293T cells, respectively (Fig. 6A). Our results showed that it was the CC domain of ITSN1-S that specifically interacted with NDH II, and the NH2-terminal (NT) domain of NDH II specifically interacted with ITSN1-S (Fig. 6B). Next, cells overexpressing 3×flag-labeled ITSN1-S with deletion of CC domain were constructed, named as shITSN1-S-3×flag-ITSN1-S-△CC/MDA-MB-231, shITSN1-S-3×flag-ITSN1-S-WT/MDA-MB-231 cells was designed as control (Fig. 6C). Disruption of the interaction of ITSN1-S and NDH II partially reversed nuclear ITSN1-S-related proliferation suppression (Fig. 6D, E), and DNA replication was accelerated in shITSN1-S-3×flag-ITSN1-S-△CC/MDA-MB-231 cells compared with the control cells (Fig. 6F). Nascent DNA abundance in shITSN1-S-3×flag-ITSN1-S-△CC/MDA-MB-231 cells was higher than control cells (Fig. 6G). We also got similar results by using KOITSN1/MDA-MB-231 cells (Fig. 6H–J). NDH II has been reported to resolve DNA–RNA hybrids and increase R-loops formation when depleted in cells [25, 26]. Our results showed that S9.6 signals were decreased in KOITSN1–3×flag-ITSN1-S-△CC/MDA-MB-231 compared with KOITSN1–3×flag-ITSN1-S-WT/MDA-MB-231 cells without RNase H pretreatment (Fig. 6K). It indicated that the ITSN1-S/NDH II interaction in nuclear may decrease the availability of NDH II at R-loops resolution, leading to an inhibition of DNA replication and nascent DNA synthesis. Altogether, these results suggested that the interaction between the CC domain of ITSN1-S and the NT domain of NDH II directly suppressed the DNA replication and nascent DNA synthesis by inhibiting R-loops resolution in breast cancer cells.

### The interaction between the EH domains of ITSN1-S and PI3KC2α in cytoplasm inhibited breast cancer cells migration and invasion by inactivation of the PI3KC2α-AKT pathway

**Fig. 7:**
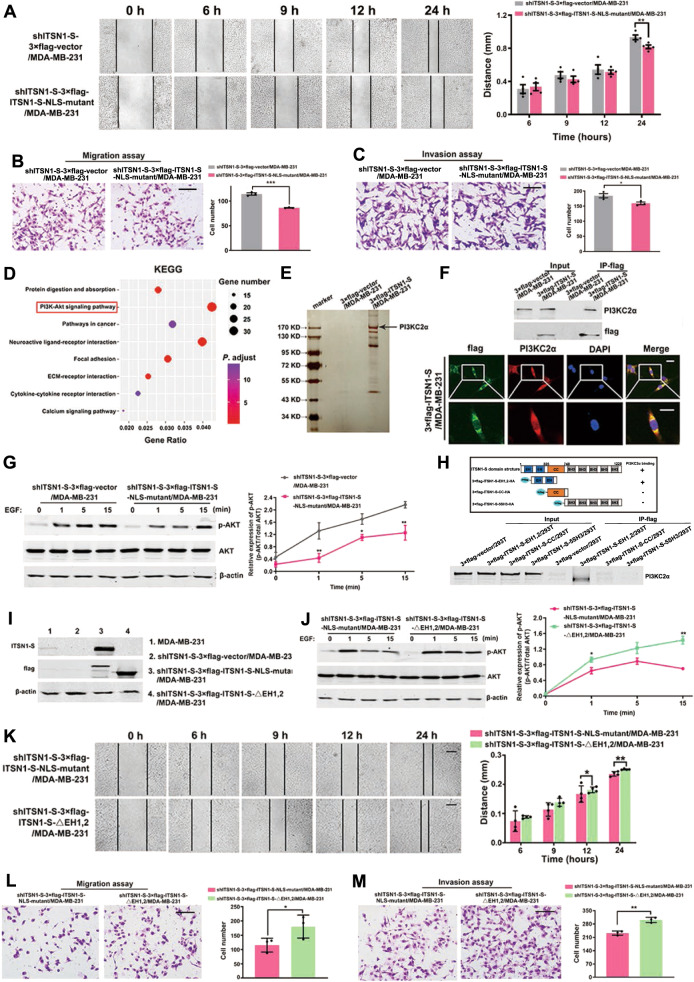
The interaction between the EH domains of ITSN1-S and PI3KC2α in cytoplasm inhibited breast cancer cells migration and invasion by inactivation of the PI3KC2α-AKT pathway. **A** Wound healing assays were performed in shITSN1-S-3×flag-vector/MDA-MB-231 and shITSN1-S-3×flag-ITSN1-S-NLS-mutant/MDA-MB-231 cells. The representative images were photographed at 0, 6, 9, 12, 24 h. Scale bars, 200 μm. Quantitative results were analyzed in the right panel. Values were expressed as mean ± SD from four independent experiments (two-tailed Student’s *t*-test and two-way ANOVA, ^*^P < 0.05, ^**^P < 0.01, ^***^P < 0.001). **B**, **C** Migration assays (**B**) and invasion assays (**C**) were performed in shITSN1-S-3×flag-vector/MDA-MB-231 and shITSN1-S-3×flag-ITSN1-S-NLS-mutant/MDA-MB-231 cells. Scale bars, 200 μm. Quantitative results were analyzed in the right panel. Values were expressed as mean ± SD from three independent experiments (two-tailed Student’s *t*-test, ^*^P < 0.05, ^**^P < 0.01, ^***^P < 0.001). **D** KEGG pathway analysis of the 759 DEGs by DAVID database. Y-axis showed the GO terms and the x-axis showed the gene ratio of each term. **E** Mass spectrometric analysis of 3×flag-ITSN1-S-associated proteins. Cellular extracts were immunopurified with anti-flag M2 gel and eluted with flag peptides. The eluates were resolved by sodium dodecyl sulfate-polyacrylamide gel electrophoresis, silver-stained, and analyzed by mass spectrometry. **F** Top: immunoprecipitation was performed with anti-flag M2 affinity gel followed by immunoblotting with antibodies against flag and PI3KC2α, respectively. Bottom: representative immunofluorescent images of the colocalization of 3×flag-labeled ITSN1-S and PI3KC2α in 3×flag-ITSN1-S/MDA-MB-231 cells. Scale bars, 25 μm. **G** shITSN1-S-3×flag-vector/MDA-MB-231 and shITSN1-S-3×flag-ITSN1-S-NLS-mutant/MDA-MB-231 cells were treated with 100 ng/ml EGF for 0, 1, 5, 15 min, the level of p-AKT was examined by Western blot. Total AKT was used as a loading control. The band intensity ratio of p-AKT vs total AKT was indicated in the right panel. Values were expressed as mean ± SEM from three independent experiments (two-tailed Student’s *t*-test, ^*^P < 0.05, ^**^P < 0.01). **H** Several exogenous different domain structure fragments of ITSN1-S were transfected into HEK-293T cells and tested with an anti-flag antibody in Western blot in the right panel. β-actin was used as a loading control. Cellular extracts of the above cells were applied, and IP experiments were performed by an anti-flag M2 affinity gel. The expression of PI3KC2α was determined by Western blot analysis in the left panel. **I** 3×flag-labeled vector, 3×flag-labeled ITSN1-S-NLS-mutant, and 3×flag-labeled ITSN1-S-△EH1,2 were transfected into shITSN1-S #2/MDA-MB-231 cells and tested with anti-flag and anti-ITSN1-S antibodies by Western blot, respectively. β-actin was used as a loading control. **J** shITSN1-S-3×flag-ITSN1-S-NLS-mutant/MDA-MB-231 and shITSN1-S-3×flag-ITSN1-S-△EH1,2/MDA-MB-231 cells were treated with 100 ng/ml EGF for 0, 1, 5, 15 min, the level of p-AKT was examined by Western blot. Total AKT was used as a loading control. The band intensity ratio of p-AKT vs total AKT is indicated in the right panel. Values were expressed as mean ± SEM from three independent experiments (two-tailed Student’s *t*-test, ^*^P < 0.05, ^**^P < 0.01). **K** Wound healing assays were performed in shITSN1-S-3×flag-ITSN1-S^-^NLS-mutant/MDA-MB-231 and shITSN1-S-3×flag-ITSN1-S-△EH1,2/MDA-MB-231 cells. The representative images were photographed at 0, 6, 9, 12, 24 h. Scale bars, 200 μm. Quantitative results were analyzed in the right panel. Values were expressed as mean ± SD from four independent experiments (two-tailed Student’s *t*-test, ^*^P < 0.05, ^**^P < 0.01). **L**, **M** Migration assays (**L**) and invasion assays (**M**) were performed in shITSN1-S-3×flag^-^ITSN1-S-NLS-mutant/MDA-MB-231 and shITSN1-S-3×flag-ITSN1-S-△EH1,2/MDA-MB-231 cells. Scale bars, 200 μm. Quantitative results were analyzed in the right panel. Values were expressed as mean ± SD from three independent experiments (two-tailed Student’s *t*-test, ^*^P < 0.05, ^**^P < 0.01).

Due to NLS-mutant ITSN1-S (cytoplasmic ITSN1-S) led to lower migration and invasion capacity than control, it suggested that ITSN1-S in the cytoplasm could inhibit breast cancer cells migration and invasion (Fig. 7A–C and Supplementary Fig. S2A, B). To investigate the mechanisms of cytoplasmic ITSN1-S in cell migration and invasion, 759 DEGs were also enriched by using the DAVID database for the Kyoto Encyclopedia of Genes and Genomes (KEGG) pathway. We found 32 genes involved in the abundance of the PI3K-Akt signaling pathway (Fig. 7D). Mass spectrometry showed that protein kinase PI3KC2α may be one of the interaction proteins of cytoplasmic ITSN1-S (Fig. 7E). Furthermore, ITSN1-S and PI3KC2α co-immunoprecipitated and co-localized in the cytoplasm of MDA-MB-231 cells (Fig. 7F). PI3KC2α is one isoform of Class II PI3Ks [27], which could lead to the activation of many intracellular signaling pathways, including AKT [28, 29]. Therefore, we explored whether ITSN1-S exerted its function through the PI3KC2α/Akt pathway in the cytoplasm. Both shITSN1-S #2/MDA-MB-231 and KOITSN1/MDA-MB-231 cell clones showed that the PI3KC2α/Akt pathway was inactivated in the cytoplasmic ITSN1-S group (Fig. 7G and Supplementary Fig. S2C). Moreover, EH domains were necessary for the interaction of ITSN1-S and PI3KC2α (Fig. 7H). Then cells overexpressing 3×flag-labeled ITSN1-S with deletion of EH domains were constructed, named as shITSN1-S-3×flag-ITSN1-S-△EH1,2/MDA-MB-231 and shITSN1-S-3×flag-ITSN1-S-NLS-mutant/MDA-MB-231 cells, which accumulated in the cytoplasm, was designed as control (Fig. 7I). After disruption of the interaction of ITSN1-S and PI3KC2α, the p-AKT expression level was restored (Fig. 7J). The migration and invasion abilities were also reversed in shITSN1-S-3×flag-ITSN1-S-△EH1,2/MDA-MB-231 cells (Fig. 7K–M). We got similar results by using ITSN1 knockout cell clones (Supplementary Fig. S2D–G). The above findings indicated that the interaction between the EH domains of ITSN1-S and PI3KC2α in cytoplasm inhibited breast cancer cells migration and invasion by inactivation of the PI3KC2α-AKT pathway.

### Combination analysis of cytoplasmic and nuclear ITSN1-S is an independent prognosis factor and patients with a high cytoplasmic ITSN1-S and a positive nuclear ITSN1-S expression had the best prognosis outcome

**Fig. 8:**
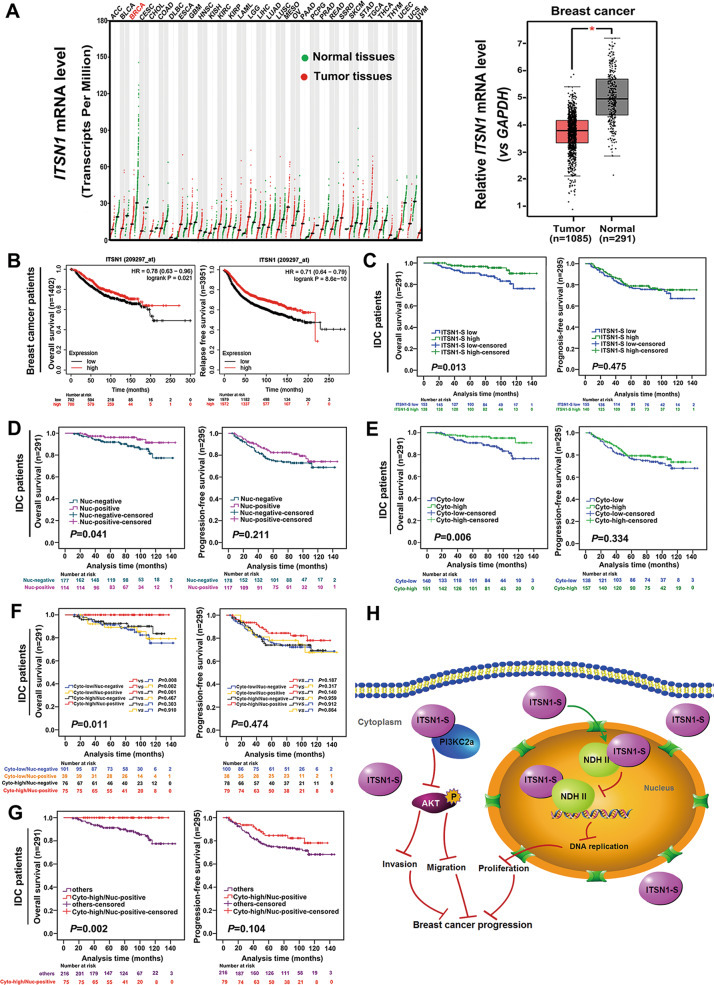
Clinical data demonstrated that patients with a high cytoplasmic ITSN1-S and a positive nuclear ITSN1-S expression had the best prognosis outcome. **A** The mRNA expression levels of *ITSN1* in multiple tumor tissues and paired normal tissues in the GEPIA database. The red dots and green dots represented tumor tissues and normal tissues, respectively. The mRNA expression levels of *ITSN1* in breast cancer tissues (*n* = 1085) and paired normal breast tissues (*n* = 291) in GEPIA database (^*^P < 0.05). **B** OS (left panel) and RFS (right panel) curves of breast cancer patients with *ITSN1* mRNA expression in Kaplan–Meier plotter database (log-rank test). **C** OS (left panel) and PFS (progression-free survival, right panel) curves of IDC patients with ITSN1-S expression (log-rank test). **D** OS (left panel) and PFS (right panel) curves of IDC patients with ITSN1-S positive nuclear expression (Nuc-positive) or ITSN1-S negative nuclear expression (Nuc-negative) (log-rank test). **E** OS (left panel) and PFS (right panel) curves of IDC patients with ITSN1-S high cytoplasmic expression (Cyto-high) or ITSN1-S low cytoplasmic expression (Cyto-low) (log-rank test). **F** OS (left panel) and PFS (right panel) curves of IDC patients with both cytoplasmic and nuclear ITSN1-S expression (log-rank test). **G** IDC patients with Cyto-high/Nuc-positive ITSN1-S expression showed a longer OS than others (log-rank test). **H** A proposed schematic model of ITSN1-S function in breast cancer progression.

The data from the GEPIA database showed that *ITSN1* mRNA level in breast cancer tissues was lower than normal tissues (Fig. 8A). ITSN1-S protein and mRNA expression levels were significantly reduced in two clones (#1 and #2, Supplementary Fig. S3A), which were used to validate the function of ITSN1-S. Reduction of ITSN1-S promoted cell growth and led to higher migration and invasion abilities (Supplementary Fig. S3B–G). The roles of ITSN1-S were also confirmed by a xenograft nude mouse model. Reduction of ITSN1-S showed a shorter survival (Supplementary Fig. S3H), and tumor volume was increased in the shITSN1-S #2/MDA-MB-231 mice group (Supplementary Fig. S3I). Deletion of ITSN1 increased cells proliferation and promoted cells migration and invasion (Supplementary Fig. S4A–C). Next, ITSN1-S expression was knocked down in T47D cells (Supplementary Fig. S5A, B). Downregulation of ITSN1-S promoted cells proliferation and invasion abilities (Supplementary Fig. S5C–E). These findings suggested that ITSN1-S may play a tumor-suppressive role in tumorigenesis of breast cancer, contrary to its function in malignant glioma in our previous studies [5, 9].

Next, survival analysis of the Kaplan–Meier-plotter database showed that breast cancer patients with higher *ITSN1* mRNA expression had a better overall survival (OS) and relapse-free survival (RFS) compared with those with lower ITSN1 mRNA expression (Fig. 8B). Consistently, our prognosis analysis showed that IDC patients with higher expression of ITSN1-S led to a longer OS (Fig. 8C). IDC patients with positive nuclear ITSN1-S expression exhibited a longer OS than patients with no nuclear ITSN1-S expression (Fig. 8D). Meanwhile, nuclear ITSN1-S expression was positively correlated with cytoplasmic ITSN1-S expression (Supplementary Table S1) and ER status (Supplementary Table S2). IDC patients with high cytoplasmic ITSN1-S expression exhibited a longer OS than those with low expression (Fig. 8E). Cytoplasmic ITSN1-S expression was negatively correlated with Ki-67 status (Supplementary Table S2). Next, we divided the cohort into four subgroups according to the subcellular expression status of ITSN1-S. The Cyto-high/Nuc-positive subgroup exhibited the longest OS compared with the other three subgroups (Fig. 8F). Then, the cohort was divided into two groups (Cyto-high/Nuc-positive and others). Consistently, the Cyto-high/Nuc-positive subgroup patients exhibited a longer OS than others (Fig. 8G). Combined cytoplasmic/nuclear ITSN1-S expression was negatively correlated with pTNM stage, lymph node metastasis status, and Ki-67 status, respectively (Supplementary Table S3). Moreover, combined analysis of cytoplasmic and nuclear ITSN1-S expression was an independent prognosis factor of breast cancer patients (Supplementary Table S4). Taken together, combination analysis of cytoplasmic and nuclear ITSN1-S is an independent prognosis factor and it is a better prognosis predictor than either alone. Finally, we summarized the signaling pathways to show our hypothesis of ITSN1-S function in breast cancer progression (Fig. 8H).

## Discussion

As an adapter protein, ITSN1-S was previously reported to localize in the cytoplasm [4, 6]. In this study, we provided for the first time that ITSN1-S was a nucleocytoplasmic trafficking protein in a CRM1-dependent pathway. Similarly, an increasing number of endocytic proteins have been shown to shuttle to the nucleus [30]. Eps15 and Epsin1, were shown to accumulate in the nucleus upon LMB treatment [31]. Other endocytic machinery members, such as Dab2, *β*-arrestins, and CALM were also proved to be nucleocytoplasmic shuttling proteins [32–35]. Moreover, several endocytic proteins were suggested to participate in transcription regulation and chromatin remodeling [36–38]. Our results strongly suggested that ITSN1-S belongs to the group of nucleocytoplasmic shuttling proteins, indicating a new direction for future research.

We found that ITSN1-S contained an NLS localized in EH domains. Then it was confirmed by mutation of basic residues of the NLS in the context of EH domains. However, mutation of the above NLS in the context of the full-length ITSN1-S protein did not completely abolish its ability to enter the nucleus, implying that there may be other unknown NLS or critical factors for ITSN1-S import into the nucleus. In addition, SH3 domains seemed to be necessary for ITSN1-S nuclear export, but we did not find a canonical NES in its SH3 domains. The complete mechanisms of ITSN1-S nucleocytoplasmic shuttling and precise functions of nuclear ITSN1-S remained to be explored.

Our present study indicated that nuclear ITSN1-S could suppress DNA replication and nascent DNA synthesis by inhibiting R-loops resolution in breast cancer cells. It is well known that R-loops cause DNA replication conflicts that commonly lead to stalled and collapsed replication forks [5]. In fact, increased R-loop as an obstacle on the DNA molecule, can stall the replication machinery and subsequently lead to DNA breaks. This represents an important source of DNA damage and a driver of genome instability [39, 40]. Defects in the expression of DNA damage repair (DDR) genes such as BRCA1/2, homologous recombination is compromised forcing cells to adopt alternative error-prone repair pathways, which are associated with high risk and a significant cause of the progression of breast cancer [41, 42]. Therefore, nuclear ITSN1-S not only exerts function in inhibiting DNA replication but also participates in the process of DDR needs further investigation in breast cancer.

Our data showed that the ITSN1-S level was downregulated in breast cancer tissues, and reduction or deletion of ITSN1-S promoted breast cancer progression. Consistently, ITSN1-S was downregulated in human lung cancer cells and tissue. Restoring ITSN1-S protein level decreased lung cancer cells proliferation and metastatic abilities [11]. However, our prior work showed that ITSN1-S promoted malignant glioma development [5, 9, 43]. Meanwhile, silencing ITSN1 expression decreased anchorage-independent growth in neuroblastoma [10]. Based on the above findings, breast cancer and lung cancer together belong to epithelial tissue-derived tumors, glioblastoma and neuroblastoma localize in the central nervous system. This demonstrated that the function of ITSN1-S may depend on the type of tissue or disease.

The clinical data demonstrated that patients with positive nuclear ITSN1-S expression exhibited a better survival compared with patients who had no nuclear ITSN1-S expression. And patients with higher ITSN1-S cytoplasmic expression had a longer survival compared with those with lower ITSN1-S cytoplasmic expression. Moreover, combination analysis of cytoplasmic and nuclear ITSN1-S is an independent prognosis factor, and patients with a high cytoplasmic ITSN1-S and a positive nuclear ITSN1-S expression had the best prognosis outcome. It showed a potential application of the combined consideration of the cytoplasmic and nuclear ITSN1-S as an independent prognosis factor.

## Materials and methods

### Microarray data

Breast cancer tissues with paired adjacent tissues from 56 patients (22 patients in Fig. 1A, B and 34 patients in Fig. 1A, 1C) were selected from the Department of Breast Cancer Pathology and Research Laboratory, Tianjin Medical University Cancer Institute and Hospital. None of the patients had received neoadjuvant chemotherapy or preoperative radiation therapy. The human materials were obtained with informed consent, and the study was approved by the Clinical Research Ethics Committee. Global LncRNA and mRNA expression profiling and data analysis for these tissues were obtained through microarray analysis by Agilent human LncRNA microarray 4×180 K gene expression data (Bioassay Laboratory of CapitalBio Corporation, Beijing, China). Genes with *P* < 0.05 and |Fold change | >1.5 were considered as DEGs.

### Patients’ clinical information

All 308 patients with IDC were women aged from 28 to 89 years (median age, 52 years). About 291 cases were included for OS analysis, excluding those without follow-up data (17 cases). About 295 cases were included for progression-free survival (PFS) analysis, excluding those without follow-up data (13 cases). During follow-up (median, 80 months; range, 12–146 months), 14 (4.5%) patients had a recurrence, 48 (15.6%) developed distant metastases (31 cases with bone metastasis, 17 with lung metastasis, 19 with liver metastasis, 5 with brain metastasis), and 34 (11.0%) patients died of breast cancer. Notably, multiple organic metastases were recorded for 15 patients. Fifty-five patients had disease progression (recurrence, distant metastasis, or death) within 5 years and 238 patients were disease-free for 5 years. None of the patients had received neoadjuvant chemotherapy or preoperative radiation therapy before surgery.

### Immunohistochemistry analysis and evaluation

IHC for ITSN1-S was performed with standard techniques by the streptavidin-peroxidase (S-P) method. Antigen retrieval was performed at 121 °C for 2 min 30 s. Sections were incubated with primary antibody against ITSN1-S overnight at 4 °C and then were incubated with a second antibody. The enzyme-substrate was 3, 3′-diaminobenzidine tetrahydrochloride (DAB). The histopathology and diagnosis in each case was confirmed independently by two pathologists in a blinded manner according to the World Health Organization criteria for the classification of breast cancer. ITSN1-S expression in the cytoplasm was evaluated according to the H score system, which was based on the staining intensity and the percentage of cells stained positively. Staining intensity was measured and scored as follows: 0 (−), no or low staining; 1 (+) definite but weak staining, 2 (++) moderate staining, and 3 (+++) intense staining. The percentage of breast cancer cells stained positively was scored as 0–100. Therefore, a total H score of cytoplasmic ITSN1-S ranged from 0 to 300 by multiplying the intensity and the percentage scores. A cytoplasmic ITSN1-S score of 0–159 as Cyto-low (low cytoplasmic expression) and a score of 160–300 was defined as Cyto-high (high cytoplasmic expression). Because nuclear staining was present in a uniform intensity but to a different extent, sections with ITSN1-S stained positively in the nuclei of breast cancer cells were defined as Nuc-positive (nuclear positive expression), while sections without ITSN1-S stained positively in the nuclei of breast cancer cells were defined as Nuc-negative (nuclear negative expression).

### Cell culture and reagents

MDA-MB-231 and HEK-293T cells were cultured in DMEM supplemented with 10% fetal bovine serum (FBS) in a 5% CO_2_ incubator at 37 °C. T47D cells were obtained from the ATCC and cultured in RPMI-1640 medium supplemented with 15% FBS in a 5% CO_2_ incubator at 37 °C. Cells were tested and authenticated in Beijing Microread Genetics Co., Ltd. (Beijing, China) by short tandem repeat profiling. All cell lines tested negative for mycoplasma contamination. Leptomycin B (LMB), a nuclear export inhibitor, was obtained from Beyotime Biotechnology (S1726, Beijing, China). Recombinant human epithelial growth factor (EGF) was obtained from PeproTech Inc (AF-100–15, NJ, USA).

### Plasmid construction and transfection

Clone of homo sapiens of full-length ITSN1-S was obtained from Gene Copoeia Inc. (CA, USA). ITSN1-S specific shRNA (#1: GATCCggtccttctcctttcacatggCTCGAGccaggaagaggaaagtgtaccTTTTTG, #2: GATCCgcagaggagttcagtatctctCTCGAGcgtctccctcaagtcatagagaTTTTTG, #3: GATCCcagctgtaccttgttgagcatCTCGAGatgctcaacaaggtacagctgTTTTTG, #4: GATCCgcatgtaatacatcctgtacaCTCGAGcgtacattatgtaggacatgtTTTTTG, #5: GATCCgctattaccttgtacgatgctCTCGAGcgataatggaacatgctacgaTTTTTG) and scrambled sequence (GATCCgttctccgaacgtgtcacgtCTCGAGacgtgacacgttcggagaacTTTTTG) were synthesized and cloned into pLVX-shRNA2-Neo lentiviral vector, respectively. The mutants of ITSN1-S and NDH II were amplified by PCR using primers (GenBank). HA and 3×flag-labeled fragments of ITSN1-S and NDH II, including ITSN1-S-EH1,2 (1–325 aa), ITSN1-S-CC (326–740 aa), ITSN1-S-5SH3 (741–1220 aa), ITSN1-S-△EH1,2 (310–1220 aa), ITSN1-S-△CC, NDH II-NT (1–380 aa), NDH II-M (381–830 aa), and NDH II-CT (831–1270 aa) were inserted into a pCDH-CMV-MCS-EF1-Puro lentiviral vector, respectively. The plasmids were next transfected into HEK-293T cells with the packing plasmids pMD2G and psPAX2 to produce lentivirus. Stable lentivirus-infected cells were selected with puromycin or G418 and verified by Western blot analysis or RT-qPCR.

### CRISPR-Cas9 deletion of ITSN1 in MDA-MB-231 cells

CRISPR-Cas9 plasmid for ITSN1 deletion was constructed with the gRNA sequence (5′-TATCTGGGCCATAACTGTAG-3′) targeting the exon 3 of ITSN1. MDA-MB-231 cells were infected with the lentivirus containing the CRISPR-Cas9 construct, and puromycin-resistant cell-derived colonies were analyzed by western blot and DNA sequencing to confirm ITSN1 deletion.

### Antibodies

Polyclonal anti-human ITSN1-S antibodies were raised in rabbits as previously described [9]. Anti-β-actin (sc-47778, Santa Cruz Biotechnology, USA), anti-DNA-RNA Hybrid [S9.6] (ENH001, Kerafast, USA), anti-histone h3.1 (RM2005, Ray Antibody Biotech), anti-flag tag (AF519-1, Beyotime Biotechnology), anti-HA tag (27573500, Roche, USA), anti-PI3KC2α (sc-365290, Santa Cruz Biotechnology, USA), anti-NDH II (sc-137232, Santa Cruz Biotechnology, USA, in Western blot and immunofluorescence assays), anti-NDH II (ab-26271, Abcam, UK, in immunopurification assays) antibodies were used.

### Western blot analysis

In brief, cells were lysed in ice-cold lysis buffer. Equal amounts of protein were loaded and separated by SDS-PAGE and then were transferred onto nitrocellulose membranes (Millipore, Billerica, MA, USA). The membranes were incubated overnight at 4 °C with the primary antibodies and were then treated with secondary antibodies (IRDye^®^800CW). Infrared signals were examined by using the Odyssey imaging system (Li-Cor Biosciences, Lincoln, NE, USA).

### RNA extraction and RT-qPCR

Total RNA was isolated from cells using Trizol reagent (Invitrogen, Carlsbad, CA, USA) according to the manufacturer’s instructions. cDNA was generated by the RTase M-MLV (Takara, Shiga-ken, Japan) as described in the manufacturer’s protocol. Quantification of ITSN1-S mRNA level was done by RT-qPCR using SYBR Green PCR Master Mix (TaKaRa, Shiga-ken, Japan), and the expression of GAPDH was used as the internal control. ITSN1-S primers used were shown in Supplementary Table S5. GAPDH primers were obtained from Sangon Biotech (B661104, Shanghai, China). Fold changes were calculated using the ΔΔCt method in Microsoft Excel.

### Immunofluorescence analysis

Cells were fixed with 4% paraformaldehyde first and then were permeabilized with 0.2% Triton X-100. The cells were incubated with primary antibodies overnight at 4 °C, and secondary antibodies (Invitrogen, New York, USA) were used at room temperature for 1 h in a dark box. Cell nuclei were stained with DAPI (Solarbio, Beijing, China), and the cells were examined by fluorescence microscopy (Carl Zeiss).

For EdU (5-Ethynyl-2′-deoxyuridine) incorporation assays, cells were washed with phosphate-buffered saline (PBS). Fresh DMEM with 10 µM EdU was then added and incubation continued for 2 h. After the incubation, fixed cells were washed with PBS and stained using the BeyoClick™ EdU Cell Proliferation Kit with Alexa Fluor 594 (C0078S, Beyotime Biotechnology) according to the manufacturer’s instructions.

S9.6 immunofluorescence was performed with some meditation as previously described [22]. Cells were fixed with 4% paraformaldehyde and then were permeabilized with 0.2% Triton X-100, blocked, and subsequently incubated with the appropriate primary and secondary antibodies. For RNase H treatments cells were incubated in the respective commercial buffers at 1× containing 60 U/ml RNase H (M0297S, NEB) for 12 h at 37 °C. Samples were then blocked, incubated with primary antibodies (ENH001, Kerafast) at room temperature overnight, followed by incubation with anti-mouse Alexa Fluor 488 (A11001, Life Technologies) secondary antibody for 1 h at room temperature.

The fluorescence intensity was measured by ImageJ software. All the immunofluorescence and microscopy experiments were performed blinded.

### Preparation of cytosol/nuclear extract

The preparation of cytosol/nuclear extract was performed essentially the same as previously described by using the Nuc-Cyto-Mem Preparation Kit (P1201, Applygen Technologies, Beijing, China) [44]. In brief, cells were lysed by Dounce homogenization with prechilled buffer cytosol extraction reagent (CER) on ice. The whole-cell lysate was then centrifuged at 800x*g* for 5 min at 4 °C. The pellet (nuclear component) was washed with the ice-cold buffer nuclear extraction reagent (NER), clarified by low-speed centrifugation, and collected as nuclei. After the supernatant of whole-cell lysate was centrifuged at 4000x*g* for 5 min at 4 °C three times, the supernatant was collected as cytoplasmic fraction. The isolated protein fractions were analyzed by Western blot.

### ATP/viability and SRB assays

Cells were plated in 24-well plates with four replicates for 5 days. Using the CellTiter-Glo Luminescent Cell Viability Assay Kit (Promega, Madison, WI, USA), ATP levels were measured as per the manufacturer’s description. For the SRB (Sulforhodamine B, Sigma, USA) assay, cells were fixed with 10% trichloroacetic acid, then were washed and stained with SRB (0.4%). Tris base (10 mM) was added to dissolve the SRB and absorbance was measured with a microplate reader at 546 nm.

### Wound healing assay

Cells were incubated in 6-well plates. When cellular density reached nearly 100%, the cell monolayer was wounded with a 20 μl micro-pipette tip. The wound areas were washed three times with PBS. Then the medium was changed to DMEM with 5% FBS. The wounds were photographed at intervals. The distance of the wounds was measured by Photoshop software.

### Migration and invasion assays

Migration and invasion assays were performed using 24-well transwell chambers (Corning, NY, USA) with polycarbonate membranes (8-μm pore size). Cells with 200 μl serum-free medium were added to the upper chamber, and 600 μl DMEM with 5% FBS was added to the lower chambers. After incubation for 24 h, cells on the upper chamber were completely scraped, and the cells on the lower surface of the membrane were fixed and stained with Giemsa solution and photographed under a microscope (Olympus, Tokyo, Japan). For invasion assay, before cells were added to the upper chamber, transwell was coated with prediluted extracellular matrix (3.2 mg/ml) (BD, NJ, USA) for 1 h. The following steps were the same as migration assay.

### Genomic and nascent DNA isolation and quantitation

Genomic DNA was isolated using the TIANamp Genomic DNA kit (TIANGEN, Being, China), as per instructions of the manufacturer, but without using RNase. Nascent DNA was prepared using the λ exonuclease method, as previously described [16], with the following modifications: The λ exonuclease-digested samples were heated at 37 °C for 12 h, then immediately subjected to electrophoreses on a 2% agarose gel. DNA was visualized by staining with 0.02% (w/v) methylene blue (Solarbio, Beijing, China) and the origin-containing nascent DNA, ranging between 350 and 1000 bp in size was excised from the gel, purified with the TaKaRa MiniBEST Agarose Gel DNA Extraction Kit (Shiga-ken, Japan), as per instructions of the manufacturer, and resuspended in RNase free water. Real-time PCR quantification analysis was performed using the LightCycler480 II (Roche, Basel, Switzerland) instrument. The sequences and amplification conditions for primer sets were shown in Supplementary Table S5. Genomic DNA from MDA-MB-231 cells was used to generate the standard curves needed for the quantification of the PCR products.

### Tumor xenograft experiments

About 5 × 10^6^ cells suspended in 200 μl PBS were injected into the third left mammary fat pad of 4-week-old female BALB/c nude mice (Model Animal Research Center of Nanjing University, Nanjing, China). When the tumor diameter reached ~1.5 cm, the tumor was used for tumor tissue block transplantation. The tumor tissues were put into physiological saline to remove necrotic tissue and then gently cut into pieces of ~8 mm^3^ on ice. Female BALB/c nude mice (4 weeks old) were randomized into different groups and used for subcutaneous transplantation. Tumor size was measured once a week by a venire caliper after the tumors formed. The minimum width (a) and ribbon width (b) of the tumor were recorded, and the tumor volume (V) was calculated as V = ab^2^/2. The total of the mice was monitored for survival. Upon death, tumor samples were obtained and fixed with 4% paraformaldehyde. Samples were soaked in wax and then were cut into 5-mm-thick sections with routine histological methods. Ki-67 staining was performed on the transplanted tumors to observe the proliferation ability of the transplanted tumors.

### Bioinformatics analysis

The breast cancer dataset including patient’s clinical information and processed RNA-sequencing data were downloaded from the database of TCGA (http://gdac.broadinstitute.org). The gene expression profiles were divided into two groups according to the median values of the expression of the *ITSN1* gene (high vs low expression). The aberrantly expressed mRNAs were analyzed by the R software. DEGs were screened by log_2_ | Fold Change | >1 and *P* < 0.05. The functional annotation tool of DAVID Bioinformatics Resources 6.8 was used to verify the remarkable enrichment of gene sets and pathways in the resulting dataset.

### Immunopurification (IP), silver staining, and mass spectrometry

These assays were performed essentially the same as previously described [45]. Cellular extracts were obtained in IP lysis buffer at 4 °C overnight followed by centrifuging at 12,000 rpm for 10 min. The protein supernatant was incubated with anti-flag M2 gel (A2220, Sigma) for 4 h at 4 °C. After washing with IP lysis buffer three times, flag peptides were applied to elute the protein complex from the gel. Final elutes were analyzed by silver staining following the manufacturer’s suggestions (P0017S, Beyotime Biotechnology). The acrylamide gel strips after silver staining were subjected to LC-MS/MS by the Beijing Genomics Institute (Beijing, China).

### CO-Immunopurification

CO-IP was performed essentially the same as previously described [45]. Cell lysates were prepared by incubating the cells in IP lysis buffer at 4 °C overnight, followed by centrifugation at 12,000x*g* for 10 min. Nonspecific protein was removed by adding Protein A (sc-2001, Santa Cruz). After the mixture was centrifuged, the supernatant was divided into two groups by using antibodies or control IgG. Finally, the precipitates were subjected to Western blot to examine the expression of target proteins.

### Two-dimensional (2D) colony formation assay

T47D cells (4 × 10^3^) were seeded into 3.5-cm dishes. After incubation at 37 °C for about 14 days, the cells were washed with PBS twice, fixed with 4% paraformaldehyde, and then stained with 0.1% crystal violet. Colonies larger than 10 μm were counted using the Olympus microscope (OLYMPUS, Japan).

### Statistical analysis

The GraphPad Prism version 6.0 and the SPSS software Version 20.0 were used for statistical analysis. The nonparametric Spearman’s correlation analysis was used to assess the association between two variables. Survival outcomes were estimated using the Kaplan–Meier method and were compared between the groups by using log-rank statistics. Univariate and multivariate Cox proportional hazards models were used to determine the associations of the clinical-pathologic parameters with survival outcomes. When comparing the means of two different groups, a two-sided Student’s *t*-test was applied. ANOVA test was performed for group comparisons. All reported *P* values were two-sided and differences reaching *P* < 0.05 were regarded as statistically significant.

## Supplementary information

Supplementary Figure S1

Supplementary Figure S2

Supplementary Figure S3

Supplementary Figure S4

Supplementary Figure S5

Supplementar figure legend

Supplementary Table S1

Supplementary Table S2

Supplementary Table S3

Supplementary Table S4

Supplementary Table S5

Supplementary Table S6

Supplementary Table S7

## Data Availability

DEGs in breast cancer tissues with paired adjacent tissues from 22 and 34 patients were included in Supplementary Table S6 and Supplementary Table S7, respectively. The mRNA expression profile of breast cancer tissues (*n* = 296) were downloaded from GEO (ID: GSE70947). The relationship between ITSN1 expression and prognosis in 1402 and 3951 breast cancer patients was analyzed by Kaplan–Meier plotter (http://www.kmplot.com/analysis/index.php?p=service&cancer=breast). KEGG pathway and GO functional enrichment analyses of 817 breast cancer patients RNA-seq data from The Cancer Genome Atlas (TCGA, https://www.cancer.gov/about-nci/organization/ccg/research/structural-genomics/tcga) was conducted using the DAVID database (https://david.ncifcrf.gov/).
