# Supplementary figures and images for: Endocytic protein intersectin1-S shuttles into nucleus to suppress the DNA replication in breast cancer
Source: Cell Death Dis. 2021 Oct 8;12(10):922. doi: 10.1038/s41419-021-04218-1 (PMC8501101; doi:10.1038/s41419-021-04218-1)

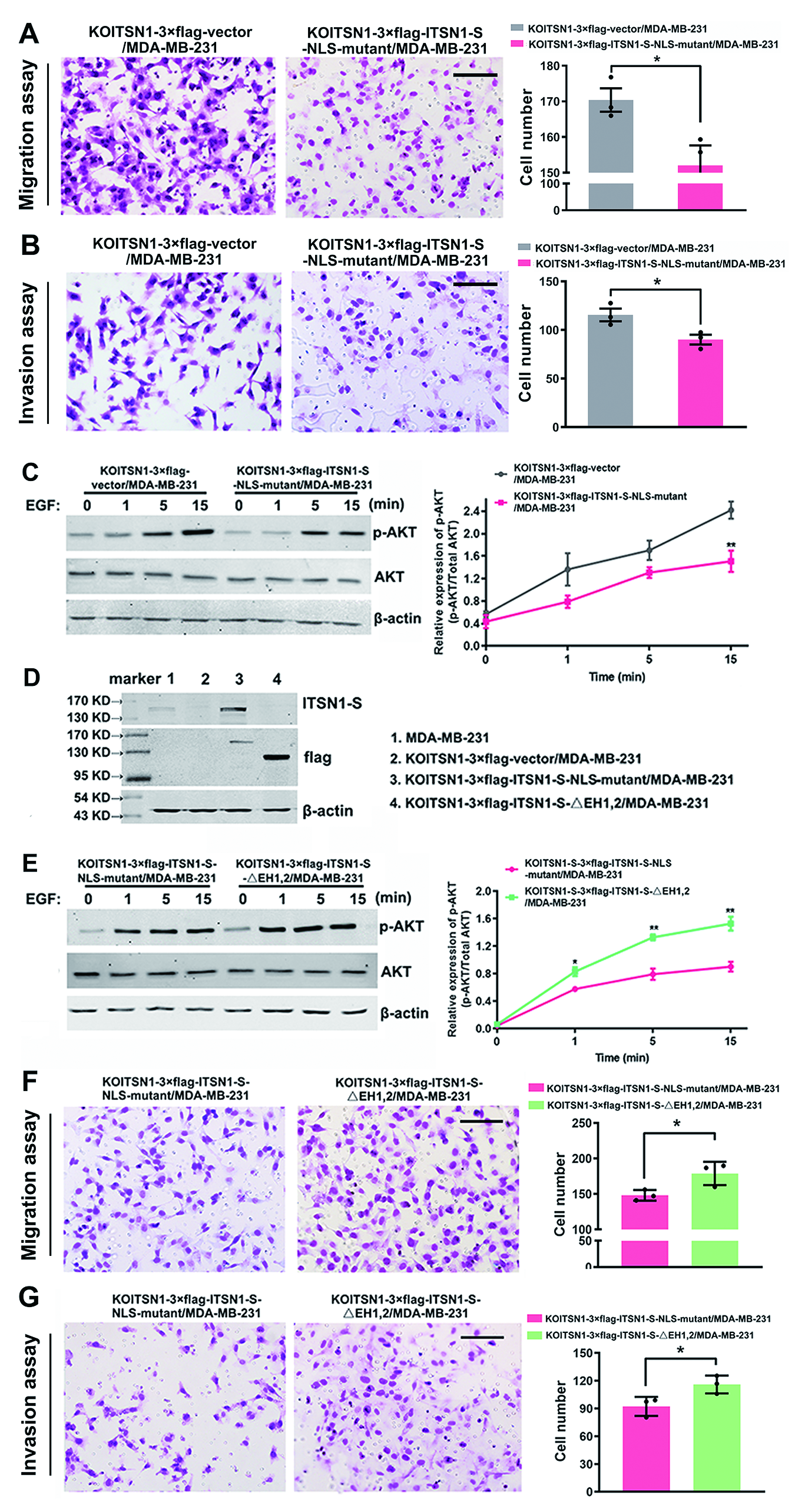

Supplement: Supplementary file 2 — Supplementary Figure S2 [file 41419_2021_4218_MOESM2_ESM.tif]

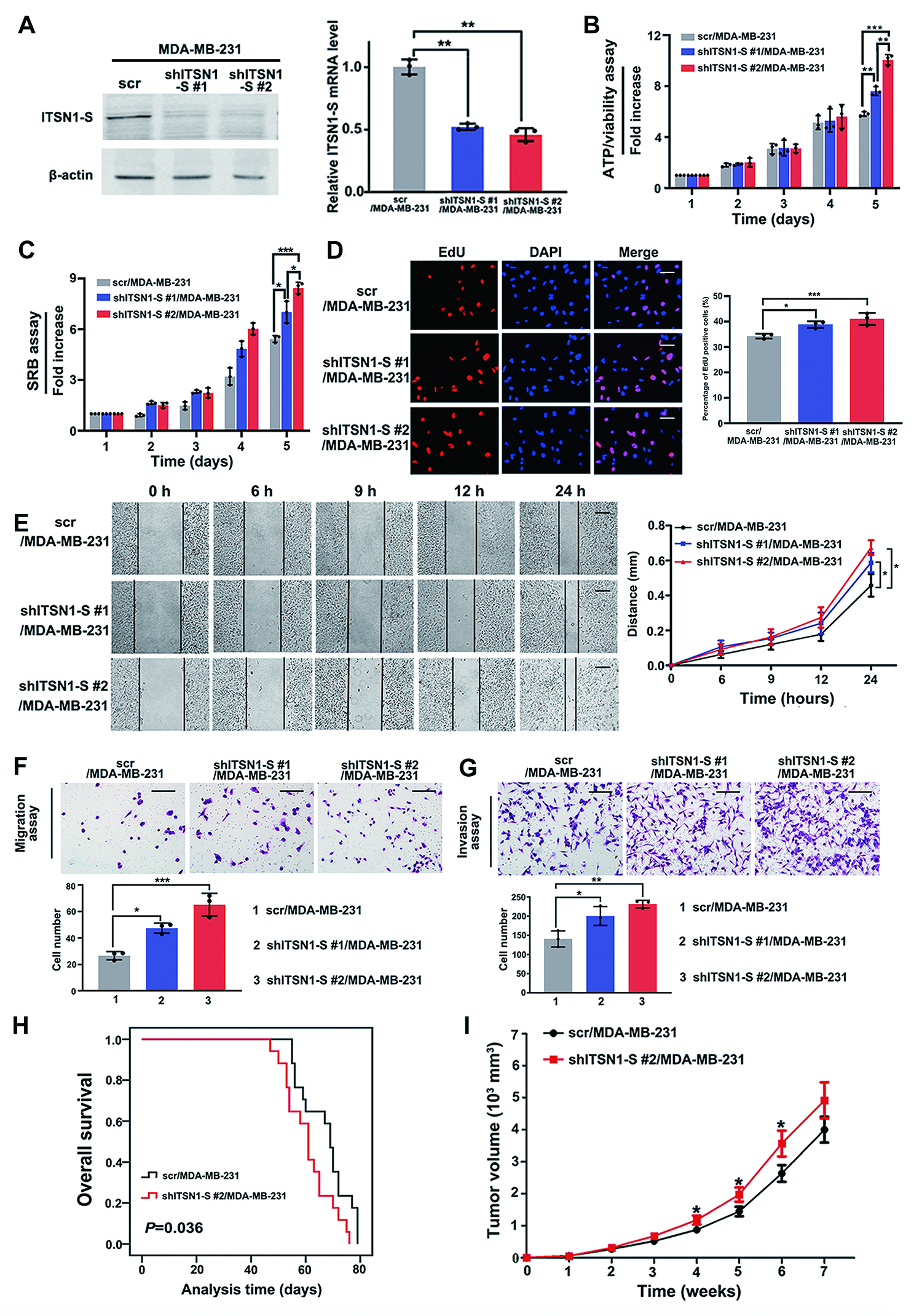

Supplement: Supplementary file 3 — Supplementary Figure S3 [file 41419_2021_4218_MOESM3_ESM.tif]

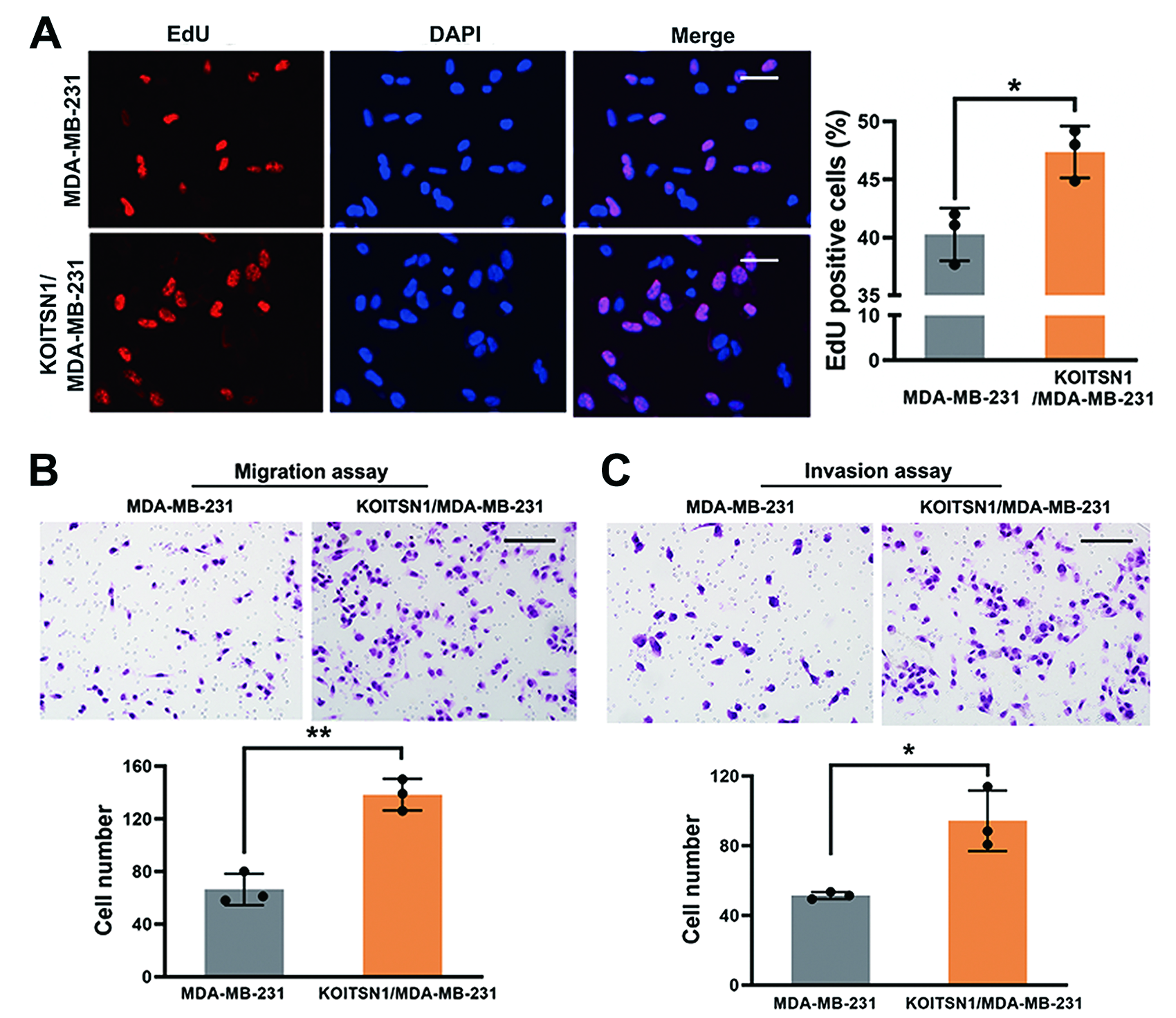

Supplement: Supplementary file 4 — Supplementary Figure S4 [file 41419_2021_4218_MOESM4_ESM.tif]

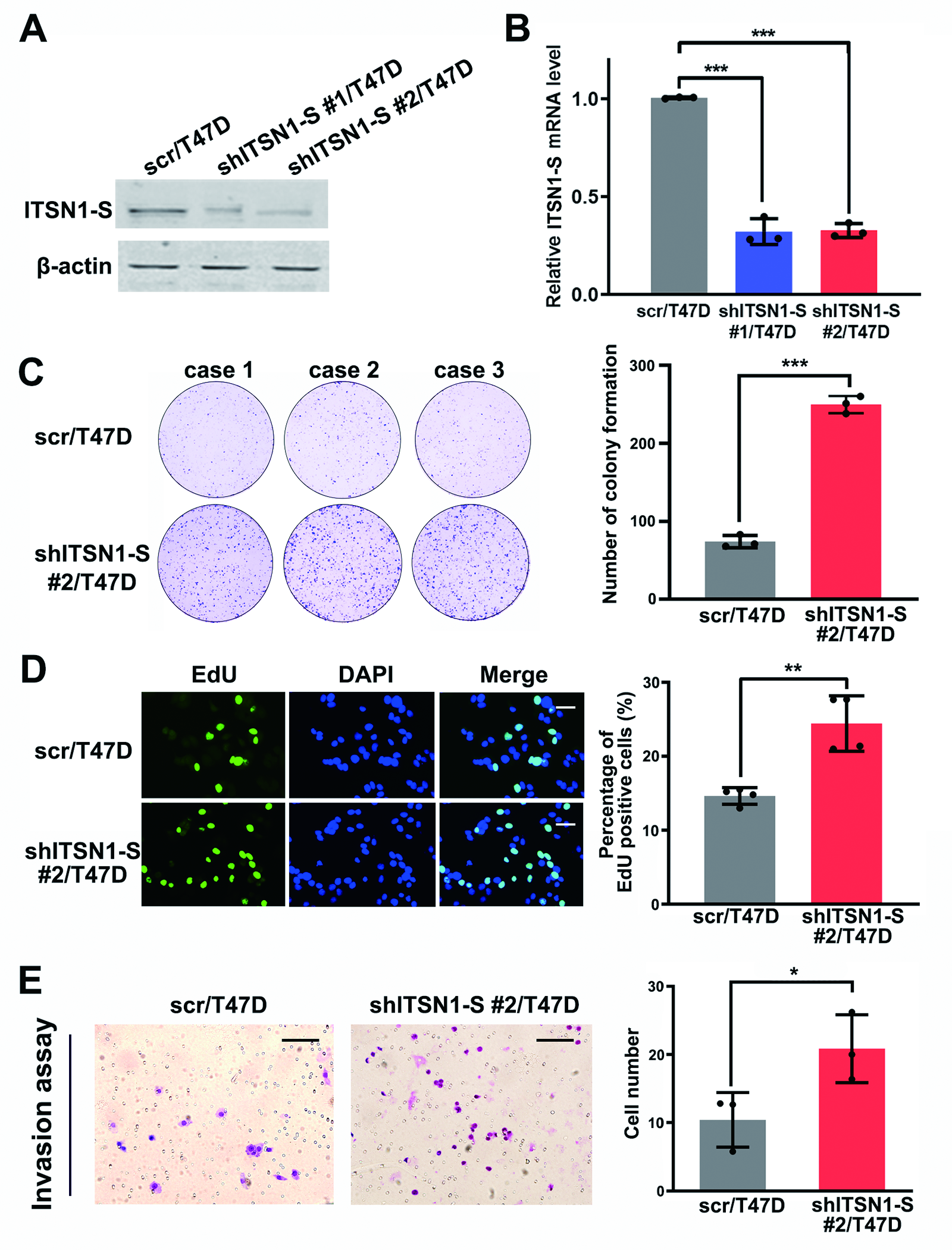

Supplement: Supplementary file 5 — Supplementary Figure S5 [file 41419_2021_4218_MOESM5_ESM.tif]
