# Supplementary material for: Endocytic protein intersectin1-S shuttles into nucleus to suppress the DNA replication in breast cancer
Source: Cell Death Dis. 2021 Oct 8;12(10):922. doi: 10.1038/s41419-021-04218-1 (PMC8501101; doi:10.1038/s41419-021-04218-1)
Supplement: Supplementary file 6 — Supplementar figure legend [file 41419_2021_4218_MOESM6_ESM.doc]

**Supplementary Figure Legends**

**Supplementary Fig. S1 Overexpression of ITSN1-S fragments in KOITSN1/MDA-MB-231 cells and knockdown of ITSN1-S in MDA-MB-231 cells. (A)** Generation of human *ITSN1* gene knockout MDA-MB-231 cells. Both sequencing and western blot confirmed the knockout of ITSN1 expression. **(B)** Expression of endogenous ITSN1-S and exogenous fragments (EH1,2, CC and 5SH3) were monitored by anti-ITSN1-S, anti-HA and anti-flag antibodies in Western blot analysis. β-actin was used as a loading control. **(C)** ITSN1-S was knocked down in MDA-MB-231 cells, and expression of ITSN1-S was determined by Western blot analysis. β-actin was used as a loading control.

**Supplementary Fig. S2 ITSN1-S in cytoplasm inhibited breast cancer cells migration and invasion by inactivation of PI3KC2α-AKT pathway. (A and B)** Migration assays **(A)** and invasion assays **(B)** were performed in KOITSN1-3×flag-vector/MDA-MB-231, KOITSN1-S-3×flag-ITSN1-S-NLS-mutant/MDA-MB-231 and KOITSN1-3×flag-ITSN1-S-WT/MDA-MB-231 cells. Scale bars, 200 μm. Quantitative results were analyzed in the lower panel. Values were expressed as mean ± SD from three independent experiments (two-tailed Student’s t test, ^*^*P*<0.05, ^**^*P*<0.01, ^***^*P*<0.001). **(C)** KOITSN1-3×flag-vector/MDA-MB-231 and KOITSN1-3×flag-ITSN1-S-NLS-mutant/MDA-MB-231 cells were treated with 100 ng/ml EGF for 0, 1, 5, 15 min, the level of p-AKT was examined by Western blot. Total AKT was used as a loading control. The band intensity ratio of p-AKT vs total AKT was indicated in the right panel. Values were expressed as mean ± SEM from three independent experiments (two-tailed Student’s t test, ^*^*P*<0.05, ^**^*P*<0.01). **(D)** 3×flag-labeled vector, 3×flag-labeled ITSN1-S-NLS-mutant and 3×flag-labeled ITSN1-S-△EH1,2 were transfected into KOITSN1/MDA-MB-231 cells and tested with anti-flag and anti-ITSN1-S antibodies by Western blot, respectively. β-actin was used as a loading control. **(E)** KOITSN1-3×flag-ITSN1-S-NLS-mutant/MDA-MB-231 and KOITSN1-3×flag-ITSN1-S-△EH1,2/MDA-MB-231 cells were treated with 100 ng/ml EGF for 0, 1, 5, 15 min, the level of p-AKT was examined by Western blot. Total AKT was used as a loading control. The band intensity ratio of p-AKT vs total AKT is indicated in the right panel. Values were expressed as mean ± SEM from three independent experiments (two-tailed Student’s t test, ^*^*P*<0.05, ^**^*P*<0.01). (**F and G**) Migration assays (**F**) and invasion assays (**G**) were performed in KOITSN1-3×flag-ITSN1-S-NLS-mutant/MDA-MB-231 and KOITSN1-3×flag-ITSN1-S-△EH1,2/MDA-MB-231 cells. Scale bars, 200 μm. Quantitative results were analyzed in the lower panel. Values were expressed as mean ± SD from three independent experiments (two-tailed Student’s t test, ^*^*P*<0.05).

**Supplementary Fig. S3 Reduction of ITSN1-S promoted proliferation, migration and invasion abilities *in vitro* and *in vivo*.** **(A)** ITSN1-S was knocked down in MDA-MB-231 cells, and expression of ITSN1-S was determined by Western blot analysis (left) and RT-qPCR analysis (right). β-actin was used as a loading control. Values were expressed as mean ± SD from three independent experiments (two-tailed Student’s t test, ^**^*P*<0.01). **(B and C)** Proliferation ability was examined by ATP/viability assay **(B)** and SRB assay **(C)** in shITSN1-S/MDA-MB-231 and control cells. Values were expressed as mean ± SD from three independent experiments (two-tailed Student’s t test, ^*^*P*<0.05, ^**^*P*<0.01, ^***^*P*<0.001). **(D)** Proliferation ability was examined by EdU incorporation assay in shITSN1-S/MDA-MB-231 and control cells. Scale bars, 25 μm. Quantitative results were analyzed in the right panel. Values were expressed as mean ± SD from three independent experiments (two-tailed Student’s t test, ^*^*P*<0.05, ^***^*P*<0.001). **(E)** Wound healing assays were performed in shITSN1-S/MDA-MB-231 and control cells. The representative images were photographed at 0, 6, 9, 12, 24 h. Scale bars, 200 μm. Quantitative results were analyzed in the right panel. Values were expressed as mean ± SD from three independent experiments (two-tailed Student’s t test, ^*^*P*<0.05). **(F and G)** Migration assays **(F)** and invasion assays **(G)** were performed in shITSN1-S/MDA-MB-231 and control cells. Scale bars, 200 μm. Quantitative results were analyzed in the lower panel. Values were expressed as mean ± SD from three independent experiments (two-tailed Student’s t test, ^*^*P*<0.05, ^**^*P*<0.01, ^***^*P*<0.001). **(H)** Orthotropic xenograft models were performed *in vivo*, the survival of scr/MDA-MB-231 mice group (n=17) and shITSN1-S #2/MDA-MB-231 mice group (n=17) was analyzed (log-rank test). **(I)** Quantitative results of the volume of xenograft tumors. Values were expressed as mean ± SEM (two-tailed Student’s t test, ^*^*P*<0.05).

**Supplementary Fig. S4 Deletion of ITSN1 promoted proliferation, migration and invasion abilities of MDA-MB-231 cells. (A)** Proliferation ability was examined by EdU incorporation assay in KOITSN1/MDA-MB-231 and MDA-MB-231 cells. Scale bars, 25 μm. Quantitative results were analyzed in the lower panel. Values were expressed as mean ± SD from three independent experiments (two-tailed Student’s t test, ^*^*P*<0.05). **(B and C)** Migration assays **(B)** and invasion assays **(C)** were performed in KOITSN1/MDA-MB-231 and MDA-MB-231 cells. Scale bars, 200 μm. Quantitative results were analyzed in the lower panel. Values were expressed as mean ± SD from three independent experiments (two-tailed Student’s t test, ^*^*P*<0.05, ^**^*P*<0.01).

**Supplementary Fig. S5 Reduction of ITSN1-S promoted proliferation and invasion abilities of T47D cells. (A-B)** ITSN1-S was knocked down in T47D cells, and expression of ITSN1-S was determined by Western blot analysis **(A)** and RT-qPCR analysis **(B)**. β-actin was used as a loading control. Values were expressed as mean ± SD from three independent experiments (two-tailed Student’s t test, ^***^*P*<0.001). **(C)** Proliferation ability was examined by colony formation assay in shITSN1-S #2/T47D and control cells. Quantitative results were analyzed in the right panel. Values were expressed as mean ± SD from three independent experiments (two-tailed Student’s t test, ^***^*P*<0.001). **(D)** Proliferation ability was examined by EdU incorporation assay in shITSN1-S #2/T47D and control cells. Scale bars, 25 μm. Quantitative results were analyzed in the right panel. Values were expressed as mean ± SD from three independent experiments (two-tailed Student’s t test, ^**^*P*<0.01). **(E)** Invasion assay was performed in shITSN1-S #2/T47D and control cells. Scale bars, 200 μm. Quantitative results were analyzed in the right panel. Values were expressed as mean ± SD from three independent experiments (two-tailed Student’s t test, ^*^*P*<0.05).
