# Supplementary material for: Endocytic protein intersectin1-S shuttles into nucleus to suppress the DNA replication in breast cancer
Source: Cell Death Dis. 2021 Oct 8;12(10):922. doi: 10.1038/s41419-021-04218-1 (PMC8501101; doi:10.1038/s41419-021-04218-1)
Supplement: Supplementary file 7 — Supplementary Table S1 [file 41419_2021_4218_MOESM7_ESM.doc]

**Supplementary Table S1. Relationship between ITSN1-S cytoplasmic expression and nuclear expression in IDC patients (n=308).**

|  | **n** | **ITSN1-S nuclear expression, n (%)** | | ***r*_s_** | ***P* value** |
| --- | --- | --- | --- | --- | --- |
|  |  | **Negative** | **Positive** |  |  |
| **ITSN1-S low cytoplasmic expression** | **146** | **107 (73.3)** | **39 (26.7)** | **0.244** | **<0.001^***^** |
| **ITSN1-S high cytoplasmic expression** | **162** | **80 (49.4)** | **82 (50.6)** |  |  |

**^***^*P*<0.001. *P* value was calculated by Spearman’s Rank-Correlation test.**
