# Supplementary material for: Endocytic protein intersectin1-S shuttles into nucleus to suppress the DNA replication in breast cancer
Source: Cell Death Dis. 2021 Oct 8;12(10):922. doi: 10.1038/s41419-021-04218-1 (PMC8501101; doi:10.1038/s41419-021-04218-1)
Supplement: Supplementary file 8 — Supplementary Table S2 [file 41419_2021_4218_MOESM8_ESM.doc]

**Supplementary Table S2. Relationship between clinicopathological characteristics and cytoplasmic/nuclear ITSN1-S expression in IDC patients (n=308).**

| **Pathological features** | **Cases** | **ITSN1-S**  **(cytoplasm), n** | | ***r_s_*** | | ***P*** | **ITSN1-S**  **(nucleus), n** | | | ***r_s_*** | ***P*** |
| --- | --- | --- | --- | --- | --- | --- | --- | --- | --- | --- | --- |
|  |  | **Low** | **High** |  |  |  | **Negative** | | **Positive** |  |  |
| **Age** |  |  |  | | **0.016** | **0.782** | |  |  | **-0.118** | **0.039*** |
| **<50** | **143** | **69** | **74** | |  |  | | **78** | **65** |  |  |
| **≥50** | **165** | **77** | **88** | |  |  | | **109** | **56** |  |  |
| **pTNM stage*^a^*** |  |  |  | | **-0.070** | **0.270** | |  |  | **-0.116** | **0.073** |
| **I** | **31** | **12** | **19** | |  |  | | **17** | **14** |  |  |
| **II** | **84** | **39** | **45** | |  |  | | **47** | **37** |  |  |
| **III-IV** | **125** | **63** | **62** | |  |  | | **84** | **41** |  |  |
| **Tumor size*^a^*** |  |  |  | | **-0.070** | **0.270** | |  |  | **-0.011** | **0.854** |
| **<2 cm** | **31** | **12** | **19** | |  |  | | **29** | **23** |  |  |
| **2-5 cm** | **84** | **39** | **45** | |  |  | | **112** | **75** |  |  |
| **>5 cm** | **125** | **63** | **62** | |  |  | | **18** | **14** |  |  |
| **Histological grade*^a^*** |  |  |  | | **0.007** | **0.901** | |  |  | **-0.070** | **0.242** |
| **I** | **7** | **2** | **5** | |  |  | | **2** | **5** |  |  |
| **II** | **222** | **110** | **112** | |  |  | | **140** | **82** |  |  |
| **III** | **51** | **23** | **28** | |  |  | | **34** | **17** |  |  |
| **LN metastasis status*^a^*** |  |  |  | | **-0.061** | **0.917** | |  |  | **-0.098** | **0.089** |
| **Negative** | **187** | **94** | **93** | |  |  | | **120** | **67** |  |  |
| **Positive** | **116** | **51** | **65** | |  |  | | **63** | **53** |  |  |
| **ER status*^a^*** |  |  |  | | **0.016** | **0.776** | |  |  | **0.151** | **0.008**** |
| **Negative** | **135** | **65** | **70** | |  |  | | **93** | **42** |  |  |
| **Positive** | **172** | **80** | **92** | |  |  | | **93** | **79** |  |  |
| **PR status*^a^*** |  |  |  | | **-0.072** | **0.207** | |  |  | **0.105** | **0.066** |
| **Negative** | **119** | **51** | **68** | |  |  | | **80** | **39** |  |  |
| **Positive** | **187** | **94** | **93** | |  |  | | **106** | **81** |  |  |
| **HER2 status*^a^*** |  |  |  | | **-0.002** | **0.970** | |  |  | **-0.093** | **0.104** |
| **- ~ +** | **224** | **106** | **118** | |  |  | | **130** | **94** |  |  |
| **++ ~ +++** | **82** | **39** | **43** | |  |  | | **56** | **26** |  |  |
| **Ki-67 status*^a^*** |  |  |  | | **-0.127** | **0.028*** | |  |  | **-0.086** | **0.138** |
| **Negative** | **54** | **18** | **36** | |  |  | | **28** | **26** |  |  |
| **Positive** | **247** | **123** | **124** | |  |  | | **155** | **92** |  |  |
| **Distant metastasis*^a^*** |  |  |  | | **-0.034** | **0.594** | |  |  | **-0.023** | **0.714** |
| **No** | **207** | **97** | **106** | |  |  | | **121** | **82** |  |  |
| **Yes** | **48** | **25** | **23** | |  |  | | **30** | **18** |  |  |

***^a^*** **Some missing data.**

**^*^*P*<0.05, ^**^*P*<0.01.**

***P* value was calculated by Spearman’s Rank-Correlation test.**
