# Supplementary material for: Endocytic protein intersectin1-S shuttles into nucleus to suppress the DNA replication in breast cancer
Source: Cell Death Dis. 2021 Oct 8;12(10):922. doi: 10.1038/s41419-021-04218-1 (PMC8501101; doi:10.1038/s41419-021-04218-1)
Supplement: Supplementary file 9 — Supplementary Table S3 [file 41419_2021_4218_MOESM9_ESM.doc]

**Supplementary Table S3. Relationship between clinicopathological characteristics and combined cytoplasmic/nuclear ITSN1-S expression status in IDC patients (n=308).**

| **Pathological features** | | **n** | **ITSN1-S cytoplasmic/nuclear expression, n (%)** | | ***r*_s_** | ***P* value** |
| --- | --- | --- | --- | --- | --- | --- |
|  |  |  | **Others** | **Cyto-high/Nuc-positive** |  |  |
| **Age, year** |  | |  |  |  |  |
| **<50** | **143** | | **103 (72.0)** | **40 (28.0)** | **-0.021** | **0.707** |
| **≥50** | **165** | | **122 (73.9)** | **43 (26.1)** |  |  |
| **pTNM stage*^a^*** |  | |  |  |  |  |
| **I** | **31** | | **20 (64.5)** | **11 (35.5)** | **-0.149** | **0.021^*^** |
| **II** | **84** | | **57 (67.9)** | **27 (32.1)** |  |  |
| **III-IV** | **125** | | **100 (80.0)** | **25 (20.0)** |  |  |
| **Histological grade*^a^*** |  | |  |  |  |  |
| **I** | **7** | | **2 (28.6)** | **5 (71.4)** | **-0.049** | **0.414** |
| **II** | **222** | | **170 (63.1)** | **52 (36.9)** |  |  |
| **III** | **51** | | **38 (74.5)** | **12 (26.5)** |  |  |
| **Tumor size, cm*^a^*** |  | |  |  |  |  |
| **<2** | **52** | | **35 (67.3)** | **17 (32.7)** | **-0.034** | **0.577** |
| **2-5** | **187** | | **135 (72.2)** | **52 (27.8)** |  |  |
| **>5** | **32** | | **23 (71.9)** | **9 (28.1)** |  |  |
| **LN metastasis status*^a^*** |  | |  |  |  |  |
| **Negative** | **187** | | **77 (66.4)** | **39 (33.6)** | **-0.116** | **0.043*** |
| **Positive** | **116** | | **144 (77.0)** | **43 (23.0)** |  |  |
| **Distant metastasis*^a^*** |  | |  |  |  |  |
| **Negative** | **203** | | **149 (73.4)** | **54 (26.6)** | **-0.033** | **0.602** |
| **Positive** | **48** | | **37 (77.1)** | **11 (22.9)** |  |  |
| **ER status*^a^*** |  | |  |  |  |  |
| **Negative** | **135** | | **104 (77.0)** | **31 (23.0)** | **0.081** | **0.156** |
| **Positive** | **172** | | **120 (69.8)** | **52 (30.2)** |  |  |
| **PR status*^a^*** |  | |  |  |  |  |
| **Negative** | **119** | | **89 (73.9)** | **31 (26.1)** | **0.013** | **0.815** |
| **Positive** | **187** | | **136 (72.7)** | **51 (27.3)** |  | |
| **Her2 status*^a^*** |  | |  |  |  | |
| **- ～ +** | **224** | | **162 (72.3)** | **62 (27.7)** | **-0.033** | **0.567** |
| **++ ～ +++** | **82** | | **62 (75.6)** | **20 (24.4)** |  |  |
| **Ki67 status*^a^*** |  | |  |  |  |  |
| **Negative** | **54** | | **32 (59.3)** | **22 (40.7)** | **-0.142** | **0.014*** |
| **Positive** | **278** | | **187 (75.7)** | **60 (24.3)** |  |  |

**Cyto: ITSN1-S cytoplasmic expression. Nuc: ITSN1-S nuclear expression.**

**Others: Cyto-high/Nuc-negative, Cyto-low/Nuc-negative and Cyto-low/Nuc-positive.**

***^a^* Some missing data.**

**^*^*P*<0.05.**

***P* value was calculated by Spearman’s rank correlation test.**
