# Supplementary material for: Endocytic protein intersectin1-S shuttles into nucleus to suppress the DNA replication in breast cancer
Source: Cell Death Dis. 2021 Oct 8;12(10):922. doi: 10.1038/s41419-021-04218-1 (PMC8501101; doi:10.1038/s41419-021-04218-1)
Supplement: Supplementary file 10 — Supplementary Table S4 [file 41419_2021_4218_MOESM10_ESM.doc]

**Supplementary Table S4. The prognosis roles of cytoplasmic and nuclear ITSN1-S expression in IDC patients.**

| **Variables** | **Univariate** | | |  | **Multivariate** | | |  | **Multivariate** | | |  | **Multivariate** | | |
| --- | --- | --- | --- | --- | --- | --- | --- | --- | --- | --- | --- | --- | --- | --- | --- |
|  | **HR** | **95%CI** | ***P* value** |  | **HR** | **95%CI** | ***P* value** |  | **HR** | **95%CI** | ***P* value** |  | **HR** | **95%CI** | ***P* value** |
| **Age** | **1.156** | **0.550-2.433** | **0.702** |  |  |  |  |  |  |  |  |  |  |  |  |
| **Tumor size** | **1.779** | **0.805-3.931** | **0.154** |  |  |  |  |  |  |  |  |  |  |  |  |
| **Histological grade** | **0.817** | **0.313-2.134** | **0.679** |  |  |  |  |  |  |  |  |  |  |  |  |
| **LN metastasis status** | **8.249** | **1.952-34.861** | **0.004**** |  | **7.951** | **1.881-33.603** | **0.005**** |  | **7.908** | **1.870-33.438** | **0.005**** |  | **8.051** | **1.906-34.005** | **0.005**** |
| **ER status** | **0.623** | **0.293-1.326** | **0.623** |  |  |  |  |  |  |  |  |  |  |  |  |
| **PR status** | **0.740** | **0.343-1.597** | **0.442** |  |  |  |  |  |  |  |  |  |  |  |  |
| **Her2 status** | **0.873** | **0.369-2.069** | **0.758** |  |  |  |  |  |  |  |  |  |  |  |  |
| **Ki-67 status** | **0.723** | **0.271-1.930** | **0.518** |  |  |  |  |  |  |  |  |  |  |  |  |
| **ITSN1-S cytoplasmic expression** | **0.318** | **0.135-0.750** | **0.009**** |  | **0.372** | **0.157-0.882** | **0.025*** |  |  |  |  |  |  |  |  |
| **ITSN1-S nuclear expression** | **0.402** | **0.163-0.992** | **0.048*** |  |  |  |  |  | **0.442** | **0.178-1.097** | **0.078** |  |  |  |  |
| **ITSN1-S cytoplasmic/nuclear expression** | **0.575** | **0.399-0.829** | **0.003*** |  |  |  |  |  |  |  |  |  | **0.591** | **0.406-0.861** | **0.006**** |

**^*^*P*<0.05, ^**^*P*<0.01.**
