# Supplementary material for: Endocytic protein intersectin1-S shuttles into nucleus to suppress the DNA replication in breast cancer
Source: Cell Death Dis. 2021 Oct 8;12(10):922. doi: 10.1038/s41419-021-04218-1 (PMC8501101; doi:10.1038/s41419-021-04218-1)
Supplement: Supplementary file 11 — Supplementary Table S5 [file 41419_2021_4218_MOESM11_ESM.doc]

| **Primer name** | **Sequence (5’-3’)** | **Size of amplicon** | **annealing temperature** |
| --- | --- | --- | --- |
| **ITSN1-S-F** | **GGGAGCCTCAAGCACAATCT** | **165 bp** | **58°C** |
| **ITSN1-S-R** | **GTRCAATGAACGAGGAGGA** |
| **Myc11-F*^a^*** | **TATCTACACTAACATCCCACGCTCTG** | **221 bp** | **66°C** |
| **Myc11-R*^a^*** | **CATCCTTGTCCTGTGAGTATAAATCATCG** |
| **Myc1-F*^a^*** | **TTCTCAACCTCAGCACTGGTGACA** | **249 bp** | **66°C** |
| **Myc1-R*^a^*** | **GACTTTGCTGTTTGCTGTCAGGCT** |

**Supplementary Table S5. Sequences and amplification conditions of primers used for real time qPCR.**

***^a^* Myc11 (peak region) and Myc1 (control region 6 kb away) are at the region of the c-*Myc* locus.**
